# ﻿A new genus *Neobelonopsis* and two new species of *Trichobelonium* (Helotiales, Ascomycota) discovered mainly from poaceous grasses native to Asia in Japan

**DOI:** 10.3897/mycokeys.99.90117

**Published:** 2023-08-14

**Authors:** Hiyori Itagaki, Tsuyoshi Hosoya

**Affiliations:** 1 Department of Botany, National Museum of Nature and Science, 4-1-1, Amakubo, Tsukuba, Ibaraki, 305-0005, Japan National Museum of Nature and Science Tsukuba Japan

**Keywords:** asexual stage, Mollisiaceae, mollisioid fungi, new taxa, phylogenetic analysis

## Abstract

Mollisioid fungi, represented by *Mollisia* (Fr.) P. Karst., are characterized by soft, sessile apothecia with globose, dark-celled excipula, hyaline ascospores, and worldwide distribution in temperate regions. Their generic and species delimitation is difficult due to the lack of distinct features, and studies based on DNA sequences are urgently required. Two genera of mollisioid fungi, *Belonopsis* and *Trichobelonium*, comprise relatively few species and are recognized by (0–)1–3-septate ascospores, medullary excipulum composed of loosely interwoven hyphae, and calcium oxalate crystals in the excipulum. Specimens of undescribed species that are morphologically assignable to *Belonopsis* or *Trichobelonium* were collected from various sites in Japan and their assignment to the proper genera was attempted. According to a molecular phylogenetic analysis involving members of Mollisiaceae based on concatenated sequences of ITS, LSU, and RPB1, eight taxonomic entities were placed in a strongly supported single clade with *Mollisiadiesbachiana*, separated from the type species of *Belonopsis*, *B.excelsior*. A new genus *Neobelonopsis* was thus proposed to accommodate the undescribed species. In this study, eight new species of *Neobelonopsis* and two new species of *Trichobelonium* were described. A new combination was also proposed for *M.diesbachiana*. The generic distinction of *Neobelonopsis* and *Trichobelonium* was supported by molecular analysis. Some additional characteristics to delimit *Trichobelonium* were identified, such as the presence of anchoring hyphae between the base of the apothecium and subiculum, and the production of abundant crystals and soluble pigments on the colonies. Derivative species of *Neobelonopsis* were found to have multi-septa in ascospores.

## ﻿Introduction

Mollisioid fungi, represented by *Mollisia* (Fr.) P. Karst. (Helotiales, Ascomycota), are characterized by soft, sessile apothecia with globose, dark-celled excipula, hyaline ascospores, and worldwide distribution in temperate regions (Nauta 2010). Not only due to the paucity of distinctive features, but also due to the presence of numerous species described with poor descriptions, the taxonomic confusion within this group has remained chaotic for a long time (Nannfeldt 1983). Johnston et al. (2019) demonstrated the monophyly of mollisioid fungi comprising at least four families of Leotiomycetes based on a multi-locus phylogenetic analysis: Mollisiaceae Rhem. (including 19 genera and >300 species), Pyrenopezizaceae Velen. (12 and >200, respectively), Drepanopezizaceae Baral (8 and >40 respectively), and Godroniaceae Baral (5 and >40, respectively) (Quandt and Haelewaters 2021). However, no consensus has been obtained regarding which family (and other related families) these mollisioid fungi should be allocated to (Baral 2016; Tanney and Seifert 2020; Wijayawardene et al. 2020).

Tanney and Seifert (2020) attempted to explore the generic boundaries within the largest family Mollisiaceae based on morphology and multigene phylogenetic analyses and proposed several nomenclatural and taxonomic options for practical treatment of the genera in this family. However, phylogenetic relationships among and within genus of mollisioid fungi have been unsolved because of the lack of authentic reference sequences and inability to obtain enough coverage for the vast number of species.

While mollisioid fungi are superficially regarded as saprophytes that form apothecia on decomposing substrates, several studies showed them as endophytes from roots and leaves of various woody plants (Sieber 1989; Shamoun and Sieber 2000; Kowalski and Andruch 2012; Tanney et al. 2016; Anderson Stewart et al. 2019; Lee et al. 2019; Itagaki and Hosoya 2021). The ecology of mollisioid fungi is more diverse than previously assumed, and it remains unclear how the ecology evolved within them. As the true species diversity and ecological evolution of mollisioid fungi may only be revealed by using DNA sequence data, the accumulation of sequences linked with apothecial morphology using voucher specimens and ecology is strongly desired (Hosoya et al. 2015).

*Belonopsis* (Sacc.) Rehm is recognized by erumpent apothecia on grasses, white to yellowish disc, brownish receptacle only at base, medullary excipulum composed of loosely interwoven hyphae with calcium oxalate crystals, and (0–)1–5-septate ascospores (Nauta and Spooner 2000). Genus *Belonopsis* is accepted by the International Code of Nomenclature for algae, fungi, and plants (Kirk et al. 2013) and is placed in Mollisiaceae by Baral (2016), but its circumscription using molecular phylogenetic analyses has not been conducted so far because DNA sequences were lacking for many species. *Trichobelonium* (Sacc.) Rehm is almost morphologically identical to *Belonopsis*, except for the well-developed subiculum and 3- to multi-septate ascospores (Rehm 1896).

Owing to the morphological similarities between *Belonopsis* and *Trichobelonium*, their distinction has been discussed for over a hundred years. *Belonopsis* was originally established as a section of *Mollisia* characterized by long ascospores, while *Trichobelonium* was proposed as a subgenus of *Belonium* Sacc. due to the presence of the subiculum (Saccardo 1889). Later, Rehm (1896) raised *Belonopsis* and *Trichobelonium* to the generic rank and distinguished *Trichobelonium* from *Belonopsis* only by the presence or absence of subiculum. In contrast, Nannfeldt (1932) claimed that the two genera were indistinguishable by the presence or absence of the subiculum, and suggested that all *Trichobelonium* species, except *T.obscurum* (Rehm) Rehm, the only one species he listed as pseudotype, could be synonymized with *Belonopsis*. Nannfeldt (1932) limited *Belonopsis* to graminicolous hosts as he believed that *Belonopsis* has a host specificity to grasses, such as Poaceae and Cyperaceae. According to Aebi (1972), the two genera are synonymous because both have filamentous ascospores with two to multiple septa and remarkable or inconspicuous subiculum. Nannfeldt (1985) observed crystal balls of calcium oxalate hydrates embedded in the medullary excipulum of several *Belonopsis* species, including species formerly placed in *Trichobelonium*, such as the type species of the genus, *T.kneiffii* (Wallr.) J. Schröt (he accidentally defined *T.obscurum* as type species in 1932). Nannfeldt (1985) suggested that the presence of crystals is an important feature in distinguishing *Belonopsis* from other genera of mollisioid fungi. Nauta and Spooner (1999 and 2000) supported the treatment of *Trichobelonium* as a synonym of *Belonopsis* and noted that future studies must determine whether Belonopsis should be considered as a subgenus of Mollisia. The former type species of synonymized *Trichobelonium*, *T.obscurum* was transferred to *Mollisia* by Richter and Baral (2008). Therefore, *Trichobelonium* has not been accepted as a valid genus, but many species still remain in this genus (Index Fungorum 2022; http://indexfungorum.org/).

Forty-two epithets in *Belonopsis* and 39 epithets in *Trichobelonium* have been listed in Index Fungorum 2022. Many species of both genera inhabit monocotyledons belonging to the families Poaceae, Cyperaceae, or Juncaceae. In Japan, *Belonopsis* and *Trichobelonium* species have not been documented except for *B.longispora* I. Hino & Katum from woody bamboo, *Pleioblastussimoni* (Hino and Katumoto 1961; Katumoto 2010). Since Japan has a rich flora of grass, we speculated that more species of *Belonopsis* and *Trichobelonium* would be found in Japan.

*Belonopsisexcelsior* (P. Karst.) Rehm, the type species of *Belonopsis*, is characterized by extremely long ascospores (42–50 μm length) with multi-septa (Rehm 1896) and has been accommodated in several genera, such as *Belonium* (Boudier 1907) and *Niptera* Fr. (Dennis 1972). Dennis (1972) transferred some species of *Belonopsis* that occur on submerged grasses to *Niptera* including *Belonopsisexcelsior* but withheld any taxonomic treatment for other terrestrial species of *Belonopsis*. Currently, Species Fungorum adopts “*Belonium*” *excelsior* (P. Karst.) Boud. However, the genus *Belonium* is also taxonomically problematic, and Baral (1994) pointed out that the generic name “*Belonium*” is used contrary to the nomenclatural rules and proposed to abandon the ambiguously used “*Belonium*” by transferring only the type species, *Beloniumgraminis* (Desm.) Sacc., to *Cejpia* Velen. (*Incertae sedis*, but closely related to Pyrenopezizaceae and Mollisiaceae), and the remaining species to *Pyrenopeziza* Fuckel. Therefore, applying “*Belonium*” to *Belonopsisexcelsior* seems inappropriate even if the species is different from other *Belonopsis* species.

In this study, we attempted to identify and classify mollisioid fungi collected mainly from poaceous grasses in Japan, based on phylogenetic analysis, morphology, and ecology (host and phenology). To assign the undescribed species to proper genera, multi-gene phylogenetic analysis was also conducted with the sequence dataset of species belonging to Mollisiaceae used by Tanney and Seifert (2020).

## ﻿Materials and methods

### ﻿Sample collection and isolation

The materials were collected from various sites in Japan. Isolates were obtained from fresh apothecia by allowing ascospores to discharge on a potato dextrose agar (PDA, Nissui, Tokyo, Japan) according to the procedure described in Itagaki et al. (2019). Germinated ascospores were transferred to a PDA slant and incubated in the dark at 20 °C. Voucher specimens were dried at 60 °C overnight and deposited at the mycological herbarium of National Museum of Nature and Science (TNS, specimens were numbered with a prefix TNS–F–). Isolates were also deposited at the Biological Resource Center, National Institute of Technology and Evaluation (**NBRC**) (Table 1).

**Table 1.** Specimens and DNA sequences used for phylogenetic analysis. All TNS specimens used in this study are in boldface. The sequences obtained form ex-type (including holo, iso, and epitype) cultures are indicated by T after the specimen/culture number.

GenBank accession No.			Specimen/Culture No.	Species name	Reference	Loacation	Host/parts
ITS	LSU	RPB1					
NR_119482	MT026532	MT018410	CBS:109321 T	*Acephalaapplanata*	Grünig et al. 2002; Tanney and Seifert 2020	Switzerland	*Piceaabies*, living root
NR_121349	MT026487	MT018414	CBS:123555 T	*Acephalamacrosclerotiorum*	Münzenberger et al. 2009; Tanney and Seifert 2020	Germany	*Pinussylvestris*, ectomycorrhizal root tip
KF874619	-	KT591690	CBS:137156 T	*Acidomelaniapanicicola*	Walsh et al. 2015	United States	*Panicumvirgatum*, root
NR_164236	-	KT591692	RUTPP WSF1R37 T	*Barreniapanicia*	Walsh et al. 2015	United States	*Panicumvirgatum*, root
NR_164237	-	KT591696	RUTPP WSF14P22 T	*Barreniataeda*	Walsh et al. 2015	United States	*Pinusrigida*, root
MH856965	MH868487	-	CBS:140.52	*Belonopsisexcelsior*	Vu et al. 2019	United Kingdom	*Phragmites*, culm
NR_119489	MH872917	MT018437	CBS:401.78 T	*Cadophoradextrinospora*	Crous et al. 2003; Tanney and Seifert 2020	Spain	Decaying wood
MH856538	MH868062	-	CBS:307.49 T	*Cadophorafastigiata*	Vu et al. 2019	Sweden	*Pinus* sp., blue-stained decaying wood
MZ159544	-	-	K(M):198911	*Cejpiahystrix*	-	United Kingdom	Unspecified
MT026425	MT026557	MT018424	CBS:295.81	*Cystodendrondryophilum*	Tanney and Seifert 2020	Switzerland	*Juniperuscommunis*, needle
MH857043	MT026562	MT018376	CBS:293.52	*Loramycesjuncicola*	Vu et al. 2019; Tanney and Seifert 2020	United Kingdom	*Eleocharispalustris*
MH857170	MT026502	MT018375	CBS:235.53 T	*Loramycesmacrosporus*	Vu et al. 2019; Tanney and Seifert 2020	United Kingdom	*Equisetumlimosum*, submerged dead culum
MT026389	MT026503	MT018366	CBS:220.56	*Mollisiacaesia*	Tanney and Seifert 2020	Netherlands	Unspecified
MT026401	MT026515	MT018353	DAOMC:251569	Mollisiacf.cinerea	Tanney and Seifert 2020	Canada	Decaying wood
MT026434	-	MT025204	DAOMC:251565	Mollisiacf.fusca	Tanney and Seifert 2020	Canada	*Betulapapyrifera*, decaying wood
MT026385	MT026496	MT018362	DAOMC:250744	Mollisiacf.melaleuca	Tanney and Seifert 2020	Canada	*Picearubens*, living needle
MT026414	MT026535	MT018415	DAOMC:250738	Mollisiacf.nigrescens	Tanney and Seifert 2020	Canada	*Picearubens*, living needle
NR171259	MT026521	MT018377	DAOMC:250732 T	*Mollisiadiesbachiana*	Tanney and Seifert 2020	Canada	Betulaalleghaniensis, decaying wood
MT026390	MT026504	MT018367	CBS:289.59	*Mollisiadiscolor*	Tanney and Seifert 2020	France	Unspecified
MT026391	MT026505	MT018368	CBS:221.56	*Mollisiafallens*	Tanney and Seifert 2020	Netherlands	Unspecified
MT026435	-	MT025205	CBS:555.63	*Mollisiafusca*	Tanney and Seifert 2020	France	*Quercus* sp.
MT026436	-	MT025208	CBS:556.63	*Mollisiahydrophila*	Tanney and Seifert 2020	France	*Phragmitesaustralis*
MT026404	MT026520	MT018378	CBS:290.59	Mollisialignivar.ligni	Tanney and Seifert 2020	France	Unspecified
MT026437	-	MT025201	CBS:291.59	Mollisialignivar.olivascens	Tanney and Seifert 2020	France	Unspecified
MT026438	-	MT025206	CBS:231.71	*Mollisialividofusca*	Tanney and Seifert 2020	Switzerland	*Loniceracoerulea*
MH861785	MT026519	MT018364	CBS:589.84	*Mollisiamelaleuca*	Vu et al. 2019	Germany	*Piceaabies*, living needle
NR171261	MT026559	MT018427	DAOMC:250734 T	*Mollisiamonilioides*	Tanney and Seifert 2020	Canada	*Picearubens*, living needle
MT026415	MT026536	MT018416	CBS:558.63	*Mollisianigrescens*	Tanney and Seifert 2020	France	Decaying wood
NR171257	MT026493	MT018359	DAOMC:252263 T	*Mollisianovobrunsvicensis*	Tanney and Seifert 2020	Canada	*Betulapapyrifera*, decaying wood
MT026440	-	MT025202	CBS:293.59	*Mollisiaolivascens*	Tanney and Seifert 2020	Unspecified	Unspecified
MT026395	MT026509	MT018372	DAOMC:251599	*Mollisiaprismatica*	Tanney and Seifert 2020	Canada	*Acersaccharum*, decaying wood
NR171260	MT026523	MT018358	DAOMC:251562 T	*Mollisiarava*	Tanney and Seifert 2020	Canada	*Betulaalleghaniensis*, rotten branch
MH860088	MT026518	MT018429	CBS:230.71	*Mollisiarosae*	Vu et al. 2019; Tanney and Seifert 2020	Italy	*Rosacanina*
MT026400	MT026514	MT018351	CBS:559.63	*Mollisiaundulatodepressula*	Tanney and Seifert 2020	France	Half submerged branch
MT026371	MT026477	MT018350	CBS:553.63	Mollisiavar.olivaecens	Tanney and Seifert 2020	France	*Betula* sp., fallen branch
MT026392	MT026506	MT018369	CBS:322.77	*Mollisiaventosa*	Tanney and Seifert 2020	Netherlands	angiosperm tree, branch
**LC682429**	**LC682462**	**LC682495**	**TNS-F-86648 T**	***Neobelonopsisacutata***	**This study**	**Japan**	***Miscanthussinensis*, decaying culum**
**LC682430**	**LC682463**	**LC682496**	**TNS-F-86671**				***Miscanthussinensis*, decaying culum**
**LC682425**	**LC682458**	**LC682491**	**TNS-F-86357**	***Neobelonopsisbicolor***			***Fraxinus* sp., decaying wood**
**LC682426**	**LC682459**	**LC682492**	**TNS-F-86605 T**				***Betula* sp., decaying wood**
**LC682427**	**LC682460**	**LC682493**	**TNS-F-86606**				***Phellodendronamurense*, decaying wood**
**LC682428**	**LC682461**	**LC682494**	**TNS-F-86664**				***Zanthoxylumailanthoides*, decaying wood**
**LC682436**	**LC682469**	**LC682502**	**TNS-F-86682 T**	***Neobelonopsiscinnabarina***			***Miscanthussinensis*, decaying culum**
**LC682437**	**LC682470**	**LC682503**	**TNS-F-86701**				***Miscanthussinensis*, decaying culum**
**LC682438**	**LC682471**	**LC682504**	**TNS-F-86716**				***Miscanthussinensis*, decaying culum**
**LC682411**	**LC682444**	**LC682477**	**TNS-F-13501 T**	***Neobelonopsisdidymospora***			**Woody bamboos, decaying culm**
**LC682412**	**LC682445**	**LC682478**	**TNS-F-13509**				***Elaeocarpusjaponicus*, decaying wood**
**LC682413**	**LC682446**	**LC682479**	**TNS-F-86178**				***Albiziajulibrissin*, decaying wood**
**LC682414**	**LC682447**	**LC682480**	**TNS-F-88720**				***Trachycarpusfortunei*, dead stem**
**LC682431**	**LC682464**	**LC682497**	**TNS-F-17105**	***Neobelonopsismicrospora***			***Sasa* sp., decaying culm**
**LC682432**	**LC682465**	**LC682498**	**TNS-F-86453**				***Sasapalmata*, decaying culm**
**LC682433**	**LC682466**	**LC682499**	**TNS-F-16804**				**Unidentified fallen branch**
**LC682434**	**LC682467**	**LC682500**	**TNS-F-18068 T**				***Sasa* sp., decaying culm**
**LC682435**	**LC682468**	**LC682501**	**TNS-F-86584**				***Sasakurilensis*, decaying culm**
**LC682415**	**LC682448**	**LC682481**	**TNS-F-61280**	***Neobelonopsismultiguttata***			***Faguscrenata, fallen cupule***
**LC682416**	**LC682449**	**LC682482**	**TNS-F-86224**				***Stephanandraincisa*, dead branche on living tree**
**LC682417**	**LC682450**	**LC682483**	**TNS-F-86402 T**				***Sasakurilensis*, decaying culm**
**LC682418**	**LC682451**	**LC682484**	**TNS-F-86465**				***Sasapalmata*, decaying culm**
**LC682420**	**LC682453**	**LC682486**	**TNS-F-15602 T**	***Neobelonopsisobtusa***			**Aucubajaponicavar.japonica, decaying wood**
**LC682421**	**LC682454**	**LC682487**	**TNS-F-44017**				**Unidentified decaying wood**
**LC682422**	**LC682455**	**LC682488**	**TNS-F-54934**				**Unidentified decaying wood**
**LC682423**	**LC682456**	**LC682489**	**TNS-F-86359**				**Fam. Lauraceae, decaying wood**
**LC682424**	**LC682457**	**LC682490**	**TNS-F-86658**				***Cornuscontroversa*, decaying wood**
**LC682419**	**LC682452**	**LC682485**	**TNS-F-86030 T**	***Neobelonopsisramosa***			***Sasa* sp., decaying culm**
MH872998	MT026501	MT018373	CBS:553.79	*Obtectodiscusaquaticus*	Vu et al. 2019; Tanney and Seifert 2020	Switzerland	*Carexrostrata*
MT026429	MT026561	MT018374	DAOMC:251536	*Ombrophilahemiamyloidea*	Tanney and Seifert 2020	Canada	Branch in stream
MT026387	MT026499	MT018412	DAOMC:251552 T	*Phialocephalaamethystea*	Tanney and Seifert 2020	Canada	*Acersaccharum*, fallen branch
NR_136124	MT026489	MT018394	DAOMC:250106 T	*Phialocephalaaylmerensis*	Tanney et al. 2016; Tanney and Seifert 2020	Canada	Decaying hardwood
MT026373	MT026482	MT018383	DAOMC:250754 T	*Phialocephalabiguttulata*	Tanney and Seifert 2020	Canada	Pinus strobus, fallen wood
NR_136122	MT026546	MT018386	DAOMC:250108 T	*Phialocephalacatenospora*	Tanney et al. 2016; Tanney and Seifert 2020	Canada	*Betulapapyrifera*, decaying branch
MT026372	MT026480	MT018381	DAOMC:250755 T	*Phialocephalacollarifera*	Tanney and Seifert 2020	Canada	*Betulapapyrifera*, decaying branch
MH862480	MT026498	MT018411	CBS:507.94 T	*Phialocephalacompacta*	Vu et al. 2019; Tanney and Seifert 2020	Germany	*Alnusglutinosa*, living bark
KP972464	MT026479	MT018380	DAOM:87232 T	*Phialocephaladimorphospora*	Tanney et al. 2016; Tanney and Seifert 2020	Canada	Pulp mill slime
AY347399	MT026526	MT018406	CBS:119271 T	*Phialocephalaeuropaea*	Grünig et al. 2002; Tanney and Seifert 2020	Switzerland	*Piceaabies*, living root
NR_103577	MT026530	MT018405	CBS:443.86 T	*Phialocephalafortinii*	Girlanda et al. 2002; Tanney and Seifert 2020	Finland	*Pinussylvestris*, root
MT026398	MT026512	MT018399	DAOMC:250756 T	*Phialocephalahelenae*	Tanney and Seifert 2020	Canada	*Acersaccharum*, fallen branch
MT026409	MT026525	MT018403	CBS:119273 T	*Phialocephalahelvetica*	Tanney and Seifert 2020	Switzerland	*Piceaabies*, living root
KP768364	MT026481	MT018382	CBS:292.59	*Phialocephalaheterosperma*	Tanney et al. 2016; Tanney and Seifert 2020	Canada	Unspecified
NR_119465	MT026538	MT018418	CBS:110521 T	*Phialocephalahiberna*	Bills 2004; Tanney and Seifert 2020	United States	*Robiniapesudoacacia*, decorticated wood
AY347391	MT026527	MT018407	CBS:119268 T	*Phialocephalaletzii*	Grünig et al. 2002; Tanney and Seifert 2020	Switzerland	*Piceaabies*, living root
NR_136123	MT026544	MT018384	DAOMC:250112 T	*Phialocephalamallochii*	Tanney et al. 2016; Tanney and Seifert 2020	Canada	Alnusalnobetulasubsp.crispa, decaying wood
NR_136121	MT026548	MT018389	DAOMC:250115 T	*Phialocephalanodosa*	Tanney et al. 2016; Tanney and Seifert 2020	Canada	*Acersaccharum*, decaying branch
KP768373	MT026552	MT018393	DAOMC:250117	*Phialocephalaoblonga*	Tanney et al. 2016; Tanney and Seifert 2020	Canada	*Betulaalleghaniensis*, decaying wood
MT026396	MT026510	MT018401	DAOMC:250101	*Phialocephalapiceae*	Tanney and Seifert 2020	Canada	*Acersaccharum*, fallen branch
NR_119460	MT026556	MT018432	CBS:468.94 T	*Phialocephalascopiformis*	Grünig et al. 2002; Tanney and Seifert 2020	Germany	*Piceaabies*, living bark
MT026411	MT026529	MT018404	CBS:134513	*Phialocephalasubalpina*	Tanney and Seifert 2020	Finland	*Pinussylvestris*, root
-	MT026531	MT018409	CBS:119234 T	*Phialocephalaturicensis*	Duó et al. 2012; Tanney and Seifert 2020	Switzerland	*Piceaabies*, living root
MT026410	MT026528	MT018408	CBS:119277 T	*Phialocephalauotilensis*	Tanney and Seifert 2020	Switzerland	*Piceaabies*, living root
MT026374	MT026483	MT018396	DAOMC:229535	*Phialocephalavermiculata*	Tanney and Seifert 2020	Canada	*Piceaglauca*, living needle
MH858062	-	MT025211	CBS:312.61	*Tapesiacinerella*	Vu et al. 2019; Tanney and Seifert 2020	France	*Fagussylvatica*, timber
MT026412	MT026533	MT018420	CBS:233.71	*Tapesiahydrophila*	Tanney and Seifert 2020	Switzerland	*Phragmitesaustralis*
MH860087	-	MT025203	CBS:228.71	*Tapesiavillosa*	Tanney and Seifert 2020	Switzerland	*Alnusalnobetula*
**LC682443**	**LC682476**	**LC682509**	**TNS-F-86430 T**	***Trichobeloniumalbobarbatum***	**This study**	**Japan**	**grass (Poaceae), decaying culm**
**LC682439**	**LC682472**	**LC682505**	**TNS-F-17835 T**	***Trichobeloniummiscanthi***			***Miscanthussinensis*, decaying culum**
**LC682440**	**LC682473**	**LC682506**	**TNS-F-30037**				***Miscanthussinensis*, decaying culum**
**LC682441**	**LC682474**	**LC682507**	**TNS-F-86672**				***Miscanthussinensis*, decaying culum**
**LC682442**	**LC682475**	**LC682508**	**TNS-F-86700**				***Miscanthussinensis*, decaying culum**
MT026474	-	-	DAOM:56173	*Trichobeloniumobscurum*	Tanney and Seifert 2020	Sweden	*Callunavulgaris*
MT026430	MT026563	MT018435	CBS:121003	*Vibrisseaflavovirens*	Tanney and Seifert 2020	Germany	*Salixalba*, branch
MT026377	MT026486	MT018434	CBS:258.91	*Vibrisseatruncorum*	Tanney and Seifert 2020	Canada	*Populus* sp., submerged root

### ﻿Morphological observations

To observe the colony morphology, mycelia grown on PDA slants were transferred to 9 cm Petri dishes containing PDA, cornmeal agar (CMA, Nissui), or 2% malt extract agar (MEA, BactoTM malt extract 20 g, agar 20 g, and water 1 L). The inoculated plates were sealed with Parafilm (Bemis, Neenah, USA) and incubated for 1–3 months at 20 °C under black light (FL15BLB, peak wavelength 352 nm, Toshiba, Tokyo, Japan). The overall appearance of the colony on PDA was photographed with a digital camera (D40, Nikon Inc., Tokyo, Japan). To observe the hyphal or conidia producing structure, mycelia were picked from the colonies using a sterilized needle, mounted in cotton blue in lactic acid (CB/LA) or water on a slide glass, and gently squashed with a cover glass.

The overall appearance of apothecia was observed under a stereomicroscope (SZ61, Olympus, Tokyo, Japan) and photographed with a digital camera (DS-L4, Olympus). To observe the pigment dissolution and discoloration of apothecia in potassium hydroxide (KOH) solution, the apothecia were immersed in 3% KOH droplets and observed under stereomicroscope.

To prepare the cross section of the apothecia, a dried apothecium was rehydrated in water, embedded in mucilage (Tissue Tek II, Miles Laboratories, Inc., Naperville, USA), and sliced at a thickness of 20–30 µm using a microtome (FX-801, Yamato Kouki, Miyazaki, Japan) equipped with an electric freezer (MC-802A, Yamato Kouki). The sections were mounted in lactic acid (LA), Melzer’s reagent (MLZ) with or without 3% KOH pretreatment, CB/LA, or water on a slide glass; examined under an optical microscope (Olympus BX51 microscope equipped with Nomarski phase interference, Olympus); and photographed with a digital camera (DS-L3, Nikon).

The length and width of 20 ascospores and 10 asci and paraphyses (from apical to second or third cell) were measured in CB/LA preparations using an ocular micrometer. Measurement of ascospores, asci, and paraphyses was conducted using rehydrated specimens. The mean ± standard deviation of each measured value with outliers in brackets is shown. Illustrations were prepared using line-drawing attachments (U-DA, Olympus). The colors of the apothecia and colonies were described by citing the codes in the CMYK systems using a color chart (DIC Corp., Tokyo). Morphological observation of microstructures of apothecium was conducted using both dried and fresh materials. When noteworthy vital reaction or distinct morphology were observed in the living materials, they were additionally described.

### ﻿Host Identification

To identify the host tree of lignicolous species, thin hand section slices of wood tissue were obtained from transversal, tangential, and radial sections using a razor blade. The sections were immersed in water for a few minutes and permanently mounted with Hoyer’s medium (Kenis, Osaka, Japan). An in-depth observation of the sliced wood tissues was performed using an optical microscope. Host tree was identified by referring to the wood identification database (https://db.ffpri.go.jp/WoodDB/IDBK-E/home.php).

### ﻿DNA extraction, PCR amplification, and sequencing

DNA was extracted from mycelia cultured in 2% malt extract broth for 2–4 weeks following the protocol previously described (Itagaki et al. 2019). Following the phylogenetic analysis by Tanney and Seifert (2020), the internal transcribed spacers (ITS1 and ITS2) and 5.8S ribosomal regions (ITS-5.8S rDNA), partial large subunit nuclear ribosomal RNA gene (LSU) and largest subunit of the nuclear RNA polymerase II gene (RPB1) regions were also determined by polymerase chain reaction (PCR) and sequencing using the following primer pairs; ITS1F and ITS4 (White et al. 1990) for ITS-5.8S rDNA, LR0R/LR5 (Vilgalys and Hester 1990; Hopple 1994) for LSU, and RPB1-Af/RPB1-6R1asc (Stiller and Hall 1997; Hofstetter et al. 2007) for RPB1. The PCR master mix contained the following reagents: 1 µL of extracted DNA, 3.5 µL of DNA-free water, 5 µL of EmeraldAmp PCR Master Mix (Takara Bio, Kusatsu, Japan), and 0.25 µL each of the forward/reverse primers. All gene regions were amplified using the following PCR cycling conditions: initial denaturation at 94 °C for 3 min, 35 cycles at 94 °C for 35 sec, 51 °C for 30 sec, and 72 °C for 60 sec, with a final extension at 72 °C for 10 min. The amplified PCR products were purified using ExoSAP-IT (Thermo Fisher Scientific, Waltham, USA) according to the manufacturer’s protocol.

Sequencing reactions were carried out using ABI PRISM 3130xl Genetic Analyzer (Applied Biosystems Inc., Norwalk, CT, USA). The obtained sequences were assembled and trimmed using the software, ATGC version 7.0.3 (Genetyx, Tokyo, Japan). The sequence data used in this study were deposited into DDBJ. The obtained ITS sequences were subjected to a Basic Local Alignment Search Tool (BLAST) search to find closely related sequences in the GenBank database.

### ﻿Taxon sampling

To examine the phylogenetic position of mollisioid fungi newly collected in Japan, the ITS, LSU, and RPB1 datasets of species in Mollisiaceae and its allies presented by Tanney and Seifert (2020) were downloaded from GenBank and included in the analysis (Table 1). The datasets consisted of ten genera in Mollisiaceae [*Acephala* Grünig & T.N. Sieber, *Barrenia* E. Walsh & N. Zhang, *Belonopsis*, *Cystodendron* Bubák, *Loramyces* W. Weston, *Mollisia*, *Obtectodiscus* E. Müll., Petrini & Samuels, *Ombrophila* Fr., *Phialocephala*, and “*Tapesia*” (Pers.) Fuckel] and *Vibrissea* Fr. in Vibrisseaceae Korf. Furthermore, ITS sequences of *Cejpiahystrix* (De Not.) Baral (=former type of *Belonium*, *B.graminis*) and “*Trichobelonium*” *obscurum* (currently transferred to *Mollisia*) were also obtained from GenBank. As outgroup, *Cadophoradextrinospora* (Korf) Koukol & Maciá-Vicente and *C.fastigiata* Lagerb. & Melin in Pyrenopezizaceae were selected.

### ﻿Phylogenetic analyses

Each region was aligned separately using MAFFT v. 7 (Katoh and Standley 2013), and all insertions/deletions were manually deleted using BioEdit ver. 7.0.5.2 (Hall 1999). The Q-INS-i option was used for ITS and LSU, and the G-INS-1 option was used for RPB1. The aligned sequences were edited manually using BioEdit. After checking no topological contradictions were observed among partitions, ITS (concatenated sequence of ITS1, 5.8S, and ITS2) dataset was analyzed using Ultrafast Maximum Likelihood (ML). All aligned genes (divided ITS, LSU, and RPB1) were automatically concatenated into a supermatrix, with sites of missing genes represented by N characters, and ITS–LSU–RPB1 concatenated dataset was analyzed by Ultrafast ML and Bayesian interface.

Ultrafast ML analysis was conducted using IQ-Tree (Nguyen et al. 2015). The automatic substitution model setting, 1,000 ultrafast bootstrap (BS) replications, and SH-aLRT branch test with 1,000 replicates was conducted by ModelFinder (Kalyaanamoorthy et al. 2017) under the Bayesian information criterion (BIC). The ML tree was made based on suitable substitution models; TIM2e+R3 for ITS1, K3P+I for 5.8S, K2P+I+G4 for ITS2, TNe+I+G4 for LSU, K3P+I+G4 for RPB1 first positions, K2P+I for RPB1 second positions, and TIM2+F+G4 for RPB1 third positions.

Bayesian inference was based on MrBayes 3.2.7a (Ronquist et al. 2012) under the most suitable substitution model for concatenated sequences were estimated using Kakusan4 (Tanabe 2011) based on the corrected BIC (Schwarz 1978). Bayesian analysis was carried out with substitution models containing the BIC4 parameter (proportional-codon-proportional model; SYM+Gamma for ITS1, K80+Gamma for 5.8S, ITS2, and LSU, GTR+Gamma for RPB1 first positions, JC69+Gamma for RPB1 second positions, and HKY85+Gamma for RPB1 third positions). The Markov chain Monte Carlo was set for four million generations with every 1,000 generations sampling except first 25% of the trees as burn-in. A 50% majority-rule consensus tree was generated, and Bayesian posterior probability (BPP) was calculated for individual branches using remaining trees.

The consensus trees were visualized using FigTree 1.4.4 (Rambaut 2018), and branches with SH-aLRT ≥80%, Ultrafast BS ≥95%, BPP ≥0.95% were regarded as strongly supported.

### ﻿BLAST search to search for closely related taxa

The obtained ITS sequences were BLAST searched to find closely related sequences in the GenBank database. If the ITS sequences of undescribed species match the existing sequences with an ≥98.5% similarity, it was discussed in the species notes.

## ﻿Results

### ﻿Molecular phylogenetic analysis

**Figure 1.**
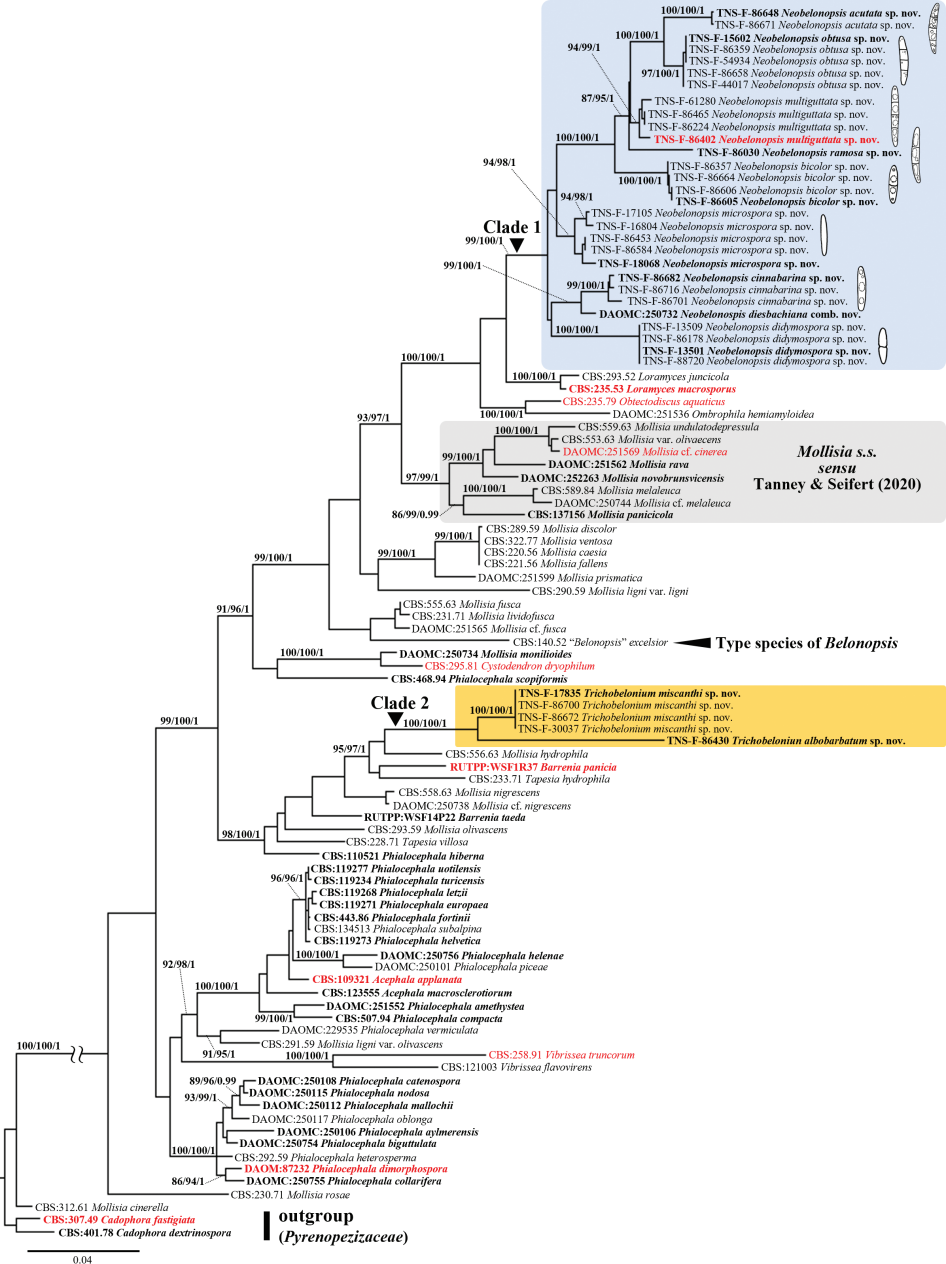
Maximum likelihood tree inferred from ITS-LSU-RPB1 concatenate sequences used in Tanney and Seifert (2020), together with sequences from additional taxa of *Neobelonopsis* and two new species of *Trichobelonium*. Significant branch supported by SH-aLRT (>80%)/Ultrafast BS (>95%)/BPP (>0.95) are indicated. Type species are in red. Collection numbers are shown at the beginning of the species name (type strains are in boldface). The tree is rooted with *Cadophoradextrinospora* and *C.fastigiata*.

ITS–LSU–RPB1 analysis included 98 taxa comprising 1,455 nucleotides, 328 from ITS, 735 from LSU, and 392 from RPB1. Since the topologies constructed using the Ultrafast ML and Bayesian analysis did not conflict with each other, only ML consensus tree is shown in Fig. 1.

**Figure 2.**
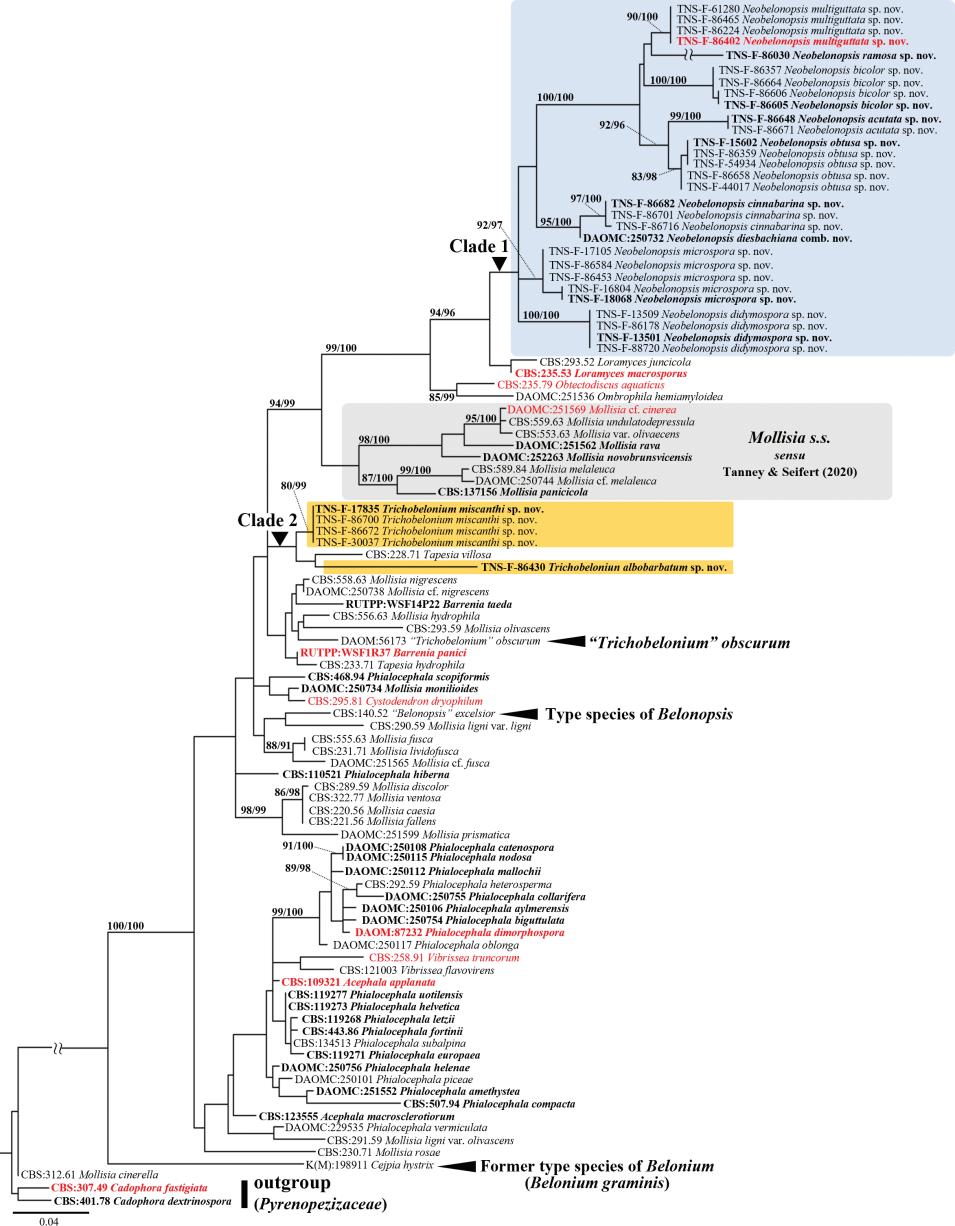
Maximum likelihood tree inferred from ITS sequences used in Tanney and Seifert (2020), together with sequences from additional taxa of *Neobelonopsis*, two new species of *Trichobelonium*, *Mollisiaobscura*, and *Cejpiahystrix*. Significant branch supported by SH-aLRT (>80%)/Ultrafast BS (>95%) are indicated. Type species are in red. Collection numbers are shown at the beginning of the species name (type strains are in boldface). The tree is rooted with *Cadophoradextrinospora* and *C.fastigiata*.

ITS phylogenetic analysis was conducted for 99 taxa (except *P.turicensis* due to lack of ITS sequence), including *Cejpiahystrix* [K(M):198911] and “*Trichobelonium*” *obscurum* (DAOM:56173) (Fig. 2). Phylogenetic placements of most species and genus in Mollisiaceae were consistent with the result of Tanney and Seifert (2020).

In the phylogenetic tree inferred from ITS–LSU–RPB1 concatenated sequence (Fig. 1), novel taxa formed two distinct clades (Clade 1 and 2) with significant support values (SH-aLRT = 99%, Ultrafast BS = 100%, and BPP = 1.00 for Clade 1, 100%, 100%, and 1.00 for Clade 2, respectively) and placed apart from “*Belonopsis*” *excelsior* (CBS:140.52) and *Mollisiasensu stricto* consisting of Mollisiacf.cinerea, M.cinereavar.olivascens (Sacc.) Boud., *M.melaleuca* (Fr.) Brunaud, *M.novobrunsvicensis* Tanney & Seifert, *M.panicicola* (E. Walsh & N. Zhang) Tanney & Seifert, *M.undulatodepressula* (Feltgen) Le Gal & F. Mangenot, *M.rava* Tanney & Seifert. Clade 1 consisted of eight taxa and *Mollisiadiesbachiana* Tanney & Seifert (DAOMC:250732), each forming strongly supported subclades. The eight taxa and *M.diesbachiana* were morphologically regarded as distinct species (see Taxonomy) and resembled *Belonopsis*, but we could not find any species of *Belonopsis* that match species collected from Japan.

Clade 1 is sister to the monophyletic genus *Loramyces*, whose generic concept differs markedly from other genera of mollisioid fungi. *Loramyces* is characterized by perithecioid apothecia surrounded by gelatinous excipulum and ascospores with gelatinous sheaths and long appendages, and suggested that divergent morphologies of apothecia and ascospores may be autapomorphic characters resulting from adaptations to aquatic or moisture environments (Weston 1929; Ingold and Chapman 1952). The present phylogenetic data supports that Clade 1 is not congeneric with *Loramyces*.

Within Clade 2, TNS-F-86430 and one monophyletic group comprising four novel taxa were found. Morphological examination (see Taxonomy) revealed two undescribed species corresponding to *Trichobelonium*. Most species placed close to Clade 2, such as *Mollisiahydrophila* (CBS:556.63, synonymy of *T.hydrophila*), *M.nigrescens* (CBS:558.63), and *T.villosa* (CBS:228.71) share a subiculum as a common feature with *Trichobelonium*, but lack septa in ascospores.

In the ML tree based on ITS sequences (Fig. 2), each novel taxon forms a strongly supported clade, but Clade 1 and 2 were weakly supported. The relationship between two undescribed species of *Trichobelonium* and “*Trichobelonium*” *obscurum* (DAOM:56173) was not resolved by ITS phylogenetic analysis. The former type of *Belonium*, *Cejpiahystrix* [K(M):198911], situated outside of Mollisiaceae.

### ﻿Taxonomy

Based on phylogenetic analyses and morphology, we proposed a new genus *Neobelonopsis* to accommodate eight new species and two new species of *Trichobelonium*. The justification for establishment of the genus and species was discussed in the following subsections. Morphologies shared by all species were described in the generic description of *Neobelonopsis* and omitted in the descriptions of each species.

#### ﻿ Neobelonopsis

Taxon classificationFungiHelotialesMollisiaceae

Itagaki & Hosoya gen. nov.

977958E8-0407-518E-A7A3-9A6130E4F002

MB843851

Figs 3 , 4 , 5 , 6 , 7 , 8 , 9 , 10 , 11 , 12 , 13 , 14

##### Etymology.

Refers to the morphological similarity with the genus, *Belonopsis*.

##### Diagnosis.

Differs from *Belonopsis* by superficial apothecia, which sometimes arise from dark-colored hyphal mass, observed as dark spots in superficial view, flattened in section (*scutum*, pl. *scuta*), wholly brownish receptacle, and the absence of crystals in the medullary excipulum. Differs from *Trichobelonium* in lacking crystals in the medullary excipulum and anchoring hyphae connecting the basal apothecia and subiculum. Differs from *Mollisia* by longer ascospores with (0–)1–3 septa, the color contrast between white hymenium and dark receptacle, and its preference for graminicolous habitats such as the culms of *Sasa* spp. and *Miscanthussinensis* Andersson.

**Figure 3.**
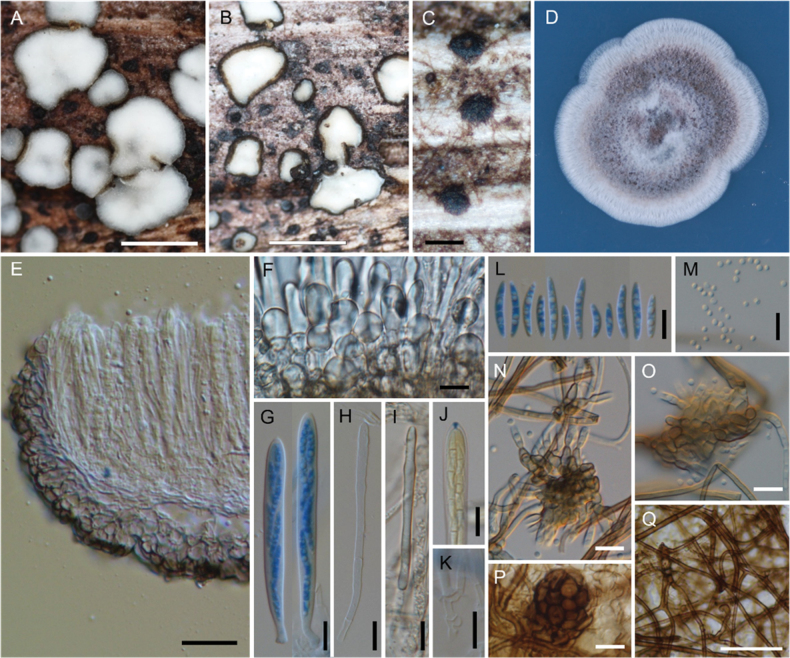
*Neobelonopsisacutata* (TNS-F-86648, holotype) **A** fresh apothecia on the decaying culm of *Miscanthussinensis***B** dried apothecia **C** stromata with sparse subiculum **D** one month old colony on PDA**E** vertical section of the apothecium (in LA) **F** refractive vacuoles in fresh marginal cells (in water) **G** asci with ascospores (in CB/LA) **H** paraphyses (in CB/LA) **I** paraphyses with a long refractive vacuole (in water) **J** blue-stained apical pore of ascus (in Melzer’s solution after 3% KOH pretreatment) **K** croziers at the base of ascus (in CB/LA) **L** ascospores (in CB/LA) **M** conidia (in water) **N, O** conidiophores (in water) **P** bulbile (in CB/LA) **Q** subicular hyphae (in CB/LA). Scale bars: 1 mm (**A, B**); 0.2 mm (**C**); 25 μm (**E, Q**); 10 μm (**F–P**).

##### Type species.

*Neobelonopsismultiguttata* Itagaki & Hosoya.

##### Description.

*Apothecia* scattered to gregarious, superficial, sometimes developed from scuta developed from poorly developed subiculum, globose to pulvinate when immature, discoid to saucer-shape when mature, flat to concave, sometimes seated on thinly subiculum, sessile, with brown to blackish receptacle; disc entire to sinuate, without hairs, waxy, often white to pale gray when fresh (rarely reddish orange), turning yellowish when dried. Ectal excipulum ***textura globulosa*** to ***angularis***, not gelatinized, without crystals or exudates, composed of 2–3 cell layers of brown thick-walled cells, brown, becoming darker toward the cortical cells; medullary excipulum ***textura intricata*** to ***prismatica***, composed of loosely interwoven hyphae, thin-walled hyphae 2–3 µm diam, hyaline. ***Asci*** cylindrical clavate, 8-spored, with a thick-walled conical apex. ***Ascospores*** ellipsoid to fusiform(-subcylindrical), with obtuse-subacute(-acute) extremes, straight to slightly curved(-sigmoid), thin-walled, 0–3(–4)-septate, with or without guttules, hyaline. ***Paraphyses*** cylindrical to slightly clavate, straight to curved, branched to simple, thin-walled, hyaline, apical cell containing long refractive vacuoles when mounted fresh in water. Conidiogenesis phialidic (resembles that of *Phialocephala* or *Cadophora*) when present.

#### ﻿ Neobelonopsis acutata

Taxon classificationFungiHelotialesMollisiaceae

Itagaki & Hosoya sp. nov.

4AB365F9-F86E-5C04-8FEC-494E9B4F6FAF

MB862636

Figs 3 , 13 , 14A

##### Etymology.

Named after the acute apices of ascospores.

##### Diagnosis.

Characterized by 3-septate ascospore with acute extremes and conidiophores densely aggregated in clusters. The present species resembles *N.multiguttata*. See Diagnosis in *N.multiguttata* for diagnostic characters.

##### Holotype.

TNS-F-86648, Yugashima, Izu City, Shizuoka Pref. Japan, 15 October 2021, on decaying culm of *Miscanthussinensis*; ex-holotype culture NBRC 115570.

##### Description.

***Apothecia*** arising from scuta. ***Scuta*** superficial, scattered to gregarious, flat discoid, blackish brown (C80M100Y80–100K60), 0.1–0.3 mm diam., ***textura epidermoidea***, composed of closely packed thick-walled cells. ***Apothecia*** 0.1–0.2 mm high, seated on subiculum, with grayish brown (C0–30M30Y40K60) to black receptacle; disc 0.25–1.4 mm diam., white to pale gray (K10) when fresh, shrunk to 0.2–1 mm diam., turns pale yellow (Y10) when dried. Ectal excipulum 25–40 µm thick at base, 15–20 µm thick at the upper flank to margin; cortical cells hemispherical to pyriform, 14–16 × 9–11 µm at base, becoming smaller to 10–12 × 7–9 µm toward the upper flank to margin, containing refractive vacuoles in the protruding cells when mounted fresh in water. Medullary excipulum 25–50 µm thick. ***Asci*** (50–)65–82(–85) × 5–9 µm, arising from croziers, with MLZ + apical pore. ***Ascospores*** 15–22(–27.5) × 2.5–3.5 µm, long fusiform, with acute apices, (1–)3(–4)-septate, containing abundant guttules, often 2–3(–4) large guttules and several smaller ones. ***Paraphyses*** (65–)70–85(–93) × 2.5–3 µm, simple, 2–3-septate, apical cells containing long refractive vacuoles when mounted fresh in water. ***Subiculum*** thinly covering the surface of substrates in patches, sparse to moderately abundant around the scuta and apothecia, shiny brown; subicular hyphae straight to curved, usually 3–5 µm diam., with 0.5–1 µm thick-walls, septate every 15–25 µm, perpendicularly branched, covered by gelatinous substance, forming bulbils of 30–45 µm across in the middle or tip, composed of densely aggregated globular or moniliform thick-walled cells, dark brown. ***Colony*** of NBRC 115570 on PDA moderately undulate, superficial, cottony to hairy, brownish gray (C20–40M40Y40K60) from the surface, zonation only observed from the reverse, without soluble pigment and crystals; aerial mycelium densely fascicular, white. ***Conidiophores*** aggregated in inconspicuous clusters on aerial hyphae, (semi-)macronematous, constricted, arising vertically or laterally from hyphae, pale to dark brown, smooth, thick-walled, frequently branched; ***phialides*** ampulliform with determinate collarettes, up to 15 µm long, approximately 4 µm width at base, discrete to integrated, terminal or intercalary, pale brown, thick-walled, with cylindrical to wide funnel-shape collarettes of 5–8 × 2.5–3 µm; ***conidia*** aseptate, spherical to subspherical, 2–2.5 µm diam., hyaline, thin-walled.

##### Additional specimen examined.

TNS-F-86671, Kawazu City, Kamo County, Shizuoka Pref., 16 October 2021, on decaying culm of *M.sinensis*, culture NBRC 115666.

##### Notes.

*Neobelonopsisacutata* resembles *Belonopsisgraminea* (P. Karst.) Sacc. & P. Syd., which has a whitish disc that turns yellowish when dried, asci, ascospores and paraphyses with overlapped biometry (Karsten 1871). However, *N.acutata* differs from *B.graminea* in its amyloid asci. *Belonopsisgraminea* produces densely aggregated conidiophores (approximately 0.2 mm across, “spermogonium” sensu Karsten) and cylindrical to elongated fusiform conidia (8–10 × 1.5 µm, “spermatia” sensu Karsten) (Karsten 1871), while *N.acutata* has sparsely aggregated conidiophores and spherical conidia (Figs 3M, 14A).

#### ﻿ Neobelonopsis bicolor

Taxon classificationFungiHelotialesMollisiaceae

Itagaki & Hosoya sp. nov.

D3B735DC-5961-51BD-8835-7F2C31328142

MB842633

Figs 4 , 13 , 14B

##### Etymology.

Named after the two-color variability observed among the apothecia in a single population.

##### Diagnosis.

Characterized by apothecia that occur only on woody substrates, 2-celled ascospores, and monilioid hyphae surrounded by a gelatinous sheath that form on artificial media.

##### Holotype.

TNS-F-86605, Kagawa Town, Muroran City, Hokkaido, Japan, 3 August 2021, on decaying wood of *Betula* sp., ex-holotype culture NBRC 115569.

##### Description.

***Apothecia*** superficial, without subiculum and scuta, 0.1–0.5 mm high, with blackish brown (C80M100Y80–100K60) to black receptacle; disc 0.8–1.5 mm diam., white to pale gray when fresh, shrunk to 0.5–1.2 mm diam., buff (M10Y30–40) or bluish gray (C30–40M20Y10–20K60) when dried. Ectal excipulum 40–50 µm thick at base, 25–40 µm thick at the upper flank to margin; cortical cells hemispherical to short clavate, 13–17 × 7.5–12 µm at base, becoming slender and smaller, moderately packed toward the margin. Medullary excipulum 10–25 µm thick, hyaline to pale brown. ***Asci*** (60–)67–80(–83) × 5–7.5 µm, arising from croziers, with MLZ + apical pore. ***Ascospores*** (10–)12–15(–17.5) × 2.5–3 µm, ellipsoid to fusiform with obtuse to subacute extremes, rarely constricted at the septum, (0–)1-septate, frequently containing two large guttules. ***Paraphyses*** (60–)62–77(–87.5) × 2.5–3(–4) µm, simple, rarely branched, 2–3-septate. ***Colony*** of NBRC 115569 on PDA convex, undulate, pulvinate, cottony to floccose, entirely pale gray (K10–40), darker from the reverse, without soluble pigment; crystals regular octahedron, 10–12.5 µm on a side, hyaline, forming on colony surface; aerial mycelium dense, white to pale gray.

##### Additional specimen examined.

TNS-F-86357, Mt. Yamizo, Daigo City, Kuji County, Ibaraki Pref., 24 May 2021, on decaying wood of *Fraxinus* sp., culture NBRC 115658; TNS-F-86606, Kagawa Town, Muroran City, Hokkaido, 3 August 2021, on decaying wood of *Phellodendronamurense*, culture NBRC 115663; TNS-F-86664, Yugashima, Izu City, Shizuoka Pref., 15 October 2021, on decaying wood of *Zanthoxylumailanthoides*, culture NBRC 115665; TNS-F-86666, Mt. Amagi, Izu City, Shizuoka Pref., 15 October 2021, on decaying wood of *Cornuscontroversa*.

**Figure 4.**
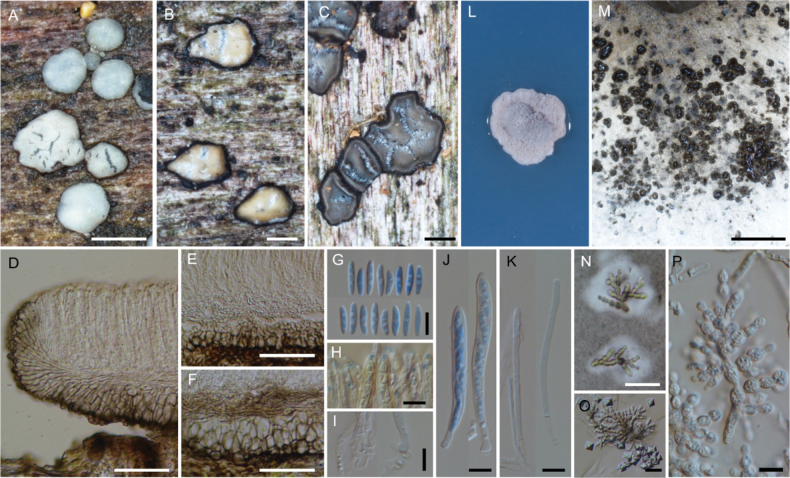
*Neobelonopsisbicolor* (TNS-F-86605, holotype) **A** fresh apothecia on decaying unidentified wood (TNS-F-86666) **B** dried apothecia with yellowish disc and blackish flask (TNS-F-86666) **C** dried apothecia with greyish discs **D** vertical section of the apothecium (TNS-F-86666, in LA) **E** hyaline medullary excipulum (TNS-F-86666, in LA) **F** brown medullary excipulum **G** ascospores (in CB/LA) **H** blue-stained apical pore of asci (in Melzer’s solution after 3% KOH pretreatment) **I** croziers at the base of asci (in CB/LA) **J** asci with ascospores (in CB/LA) **K** paraphyses (in CB/LA) **L** one month old colony on PDA**M** dark, gelatinous hyphal mass on CMA**N** monilioid hyphae surrounded by a gelatinous sheath (in diluted black ink) **O** octahedron crystals with monilioid hyphae on CMA (in water) **P** monilioid hyphae containing abundant guttles (in water). Scale bars: 1 mm (**A, M**); 0.5 mm (**B, C**); 50 μm (**D–F**); 25 μm (**N, O**); 10 μm (**G–K, P**).

##### Notes.

*Neobelonopsisbicolor* shares biometry and morphology of ascospore with *Belonopsisjuncicola* Graddon but differs in having larger asci (vs. 40 × 5 µm) and lignicolous habitat (vs. *Juncus*) (Graddon 1990).

Both TNS-F-86605 (holotype) and 86606, which were collected from the same location in Hokkaido on the same day in October, have bluish gray hymenium (Fig. 4C) and pigmented medullary excipulum (Fig. 4F). Other specimens collected from spring to summer (May to August) in Honshu (TNS-F-86357 and 86664) have whitish to yellowish hymenium (Fig. 4B) and hyaline medullary excipulum (Fig. 4E). In the phylogenetic tree (Figs 1, 2), specimens with the two-color variability of hymenium formed a well-supported identical clade. Further sampling and morphological comparisons are needed to clarify whether these morphological differences depend on geographic or seasonal variability.

*Neobelonopsisbicolor* produces dark gelatinous hyphal structures on the colony surface of CMA and 2% MEA (Fig. 4M). The hyphal structure is composed of monilioid cells hyaline to pale brown, 5–10 µm diam., containing abundant guttles and a thick-walls. The monilioid cells are arranged linearly or sympodially and branch vertically or laterally (Figs 4P, 14B). The monilioid cells are covered with a thick gelatinous sheath (Fig. 4N). No asexual stage observed in colonies on any medium.

#### ﻿ Neobelonopsis cinnabarina

Taxon classificationFungiHelotialesMollisiaceae

Itagaki & Hosoya sp. nov.

EABD8232-AE04-56AA-B3F8-D0646C313779

MB842630

Figs 5 , 13 , 14C

##### Etymology.

*Cinnabarina* in Latin, referring to the remarkable color of disc.

##### Diagnosis.

Differs from all other *Neobelonopsis* species by reddish orange disc.

##### Holotype.

TNS-F-86682, Yuzawa Town, Minami-uonuma County, Niigata Pref., Japan, 31 October 2021, on decaying culms of *Miscanthussinensis*, ex-holotype culture NBRC 115571.

##### Description.

***Apothecia*** developed from scuta. ***Scuta*** superficial, scattered to gregarious, flat discoid, pale reddish brown (C30–60M80Y80–100K10) to dark brown (C40–60M80Y100K60), 125–375 µm diam., ***textura epidermoidea***. ***Apothecia*** flat to cushion-shape, 0.2–0.5 mm high, with blackish brown (C100M100Y80–100K60) to greenish dark brown (C80M80Y80–100K60) receptacle, releasing magenta pigment (C40–20M100Y10–30K60) in 3% KOH; disc 0.6–2 mm diam., light orange (C0–30M80Y100K0) to reddish orange (C0–20M100Y100) when fresh, shrunk to 0.3–1.5 mm diam. Ectal excipulum 25–40 µm thick at base, 15–25 µm thick at the upper flask to margin; cortical cells clavate to pyriform, 14–18(–20) × 8.5–10 µm at base, becoming smaller toward the margin, 10–12 × 5–7 µm, containing yellow to orange cytoplasm which turns magenta in 3% KOH, containing guttules that disappeared in 3% KOH. Medullary excipulum 25–50 µm thick. ***Asci*** (56–)62–75(–83) × 6–7.5 µm, arising from croziers, with MLZ + apical pore. ***Ascospores*** 15–20(–22.5) × 3.5–4.5 µm, ellipsoid to subcylindrical, with rounded to subacute extremes, aseptate, hyaline or yellow when mounted fresh in water, containing 2(–4) large guttules. ***Paraphyses*** (50–)60–75(–80) × 2.5–3.5 µm, wider toward the apex up to 5 µm, simple, septum distance closer towards the base, containing long yellowish refractive vacuoles when mounted fresh in water, changed magenta in 3% KOH and showing color gradation (darker toward the tip). ***Subiculum*** thinly developed the surface of substrate, sparse to moderately abundant around the scuta and apothecia, shiny brown; subicular hyphae straight to curved, sometimes forming fascicules with 2–3 hyphae, 2.5–4 µm diam. with 0.5–1 µm thick-walls, branched at right angle, walls covered by a thick gelatinous substance. ***Colony*** of NBRC 115571 on PDA entire to slightly undulate, flat to slightly winkled, floccose to felted, brownish gray (C0–20M30–40Y40K30) from the surface, turning white at the edge, same color from the reverse, without soluble pigment; crystals ovoid to dumbbell-shape, 18–25 × 11–15 µm, hyaline, forming on surface or below agar; aerial mycelium sparse to dense, gray. ***Conidiophores*** semi-macronematous, solitary to caespitose (forming rather loose sporodochia), short, constricted, arising vertically or laterally from hyphae, pale to dark brown, smooth, thick-walled, branched; ***phialides*** round-bottom flask or bottle-shape, up to 20 µm long, 3–4 µm width at base, discrete to integrated, terminal or intercalary, pale brown, thick-walled, with cylindrical collarettes of 8–10 × 2 µm; ***conidia*** aseptate, cylindrical oblong to fusiform, abundantly aggregated in slimy heads, 4–5×1 µm, hyaline, thin-walled.

**Figure 5.**
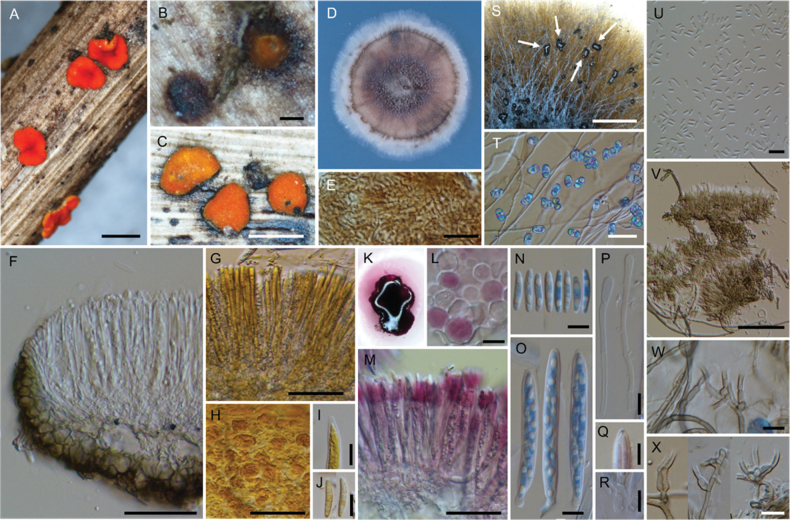
*Neobelonopsiscinnabarina* (TNS-F-86682, holotype) **A** fresh apothecia on the decaying culm of *Miscanthussinensis***B** immature apothecium protruding from the stroma **C** dried apothecia **D** one month old colony on PDA**E** texture of stroma (in LA) **F** vertical section of the apothecium (in LA) **G** yellowish reflective vacuoles in fresh paraphyses (in water) **H** yellowish vacuoles in outermost cells of fresh ectal excipulum (in water) **I** immature ascus with yellowish cytoplasm (in water) **J** ascospores with yellowish cytoplasm (in water) **K** apothecium that dissolved magenta pigments when immersed in 3% KOH**L** outermost cells that turned magenta (in 3% KOH) **M** fresh paraphyses that turned magenta, note that the tips have a darker color (in 3% KOH) **N** ascospores (in CB/LA) **O** asci with ascospores (in CB/LA) **P** paraphyses with wide, blunt head (in CB/LA) **Q** blue-stained apical pore of ascus (in Melzer’s solution after 3% KOH pretreatment) **R** crozier at the base of ascus (in CB/LA); S collapse conidiophores with slimy conidial drops on CMA (arrows) **T** hyphae with oblong-shape crystals on CMA**U** conidia (in water) **V** clusters of conidiophores (in water) **W, X** discrete conidiophores (in water). Scale bars: 1 mm (**A, S**); 0.1 mm (**B**); 0.5 mm (**C**); 50 μm (**F–H, M, T, V**); 20 μm (**E**); 10 μm (**I, J, L, N–R, U, W, X**).

##### Additional specimens examined.

TNS-F-86690 and 86692, Yuzawa Town, Minami-uonuma County, Niigata Pref., 31 October 2021, on decaying culms of *Miscanthussinensis*; TNS-F-86701, Daigenta Lake, Yuzawa Town, Minami-uonuma County, Niigata Pref., 31 October 2021, on decaying culms of *M.sinensis*, culture NBRC 115669; TNS-F-86704 and 86716 (culture NBRC 115670), Toukamachi City, Niigata Pref., 31 October 2021, on decaying culms of *M.sinensis*.

##### Notes.

*Neobelonopsiscinnabarina* is easily distinguished from other species by the reddish orange disc, slightly clavate paraphyses, and strong magenta pigment release of apothecia in KOH. In particular, the brilliant color of disc of this fungus is a rare feature in mollisioid fungi, except for *Mollisiapurpurea* Rhem and *M.russea* (Schmid-Heckel) Baral. These two species share several characters with *N.cinnabarina*, such as dark scuta [*N.russea* has “dunkelbraunen Schild” *sensu*Schmid-Heckel (1988)], bright orange vacuoles in paraphyses that become intensely magenta (red violet) in KOH, ocher to brown receptacle, asci arising from croziers, aseptate ascospores, and monocot host (Rhem 1907; Schmid-Heckel 1988; Baral and Marson 2005; Richter and Baral 2008). As the tips of fresh paraphyses turn dark magenta in 3% KOH (Fig. 5M), this phenomenon is suggested to be a vital reaction as the pigments diffuse uniformly in the paraphyses after heat drying. Richter and Baral (2008) also described the same reaction in *M.russea*. These features imply a close relationship among *N.cinnabarina*, *M.purpurea*, and *M.russea*. However, *M.russea* has no subiculum, ascospores are shorter (11–16 × 2.5–3.5 µm) than *N.cinnabarina*, and paraphyses are not clavate. *Mollisiapurpurea* also differs from *N.cinnabarina* in having crystals in medullary excipulum and shorter ascospores (12–14 × 2.5–3 µm) than *N.cinnabarina*. Genetic comparison among these species could not be conducted as *M.purpurea* and *M.russea* lack available DNA sequences.

*Neobelonopsiscinnabarina* produces conidiophores only on CMA (Fig. 5S), and conidia mostly germinate (Fig. 5U). The asexual stage of *N.cinnabarina* is unique in loose sporodochia (Fig. 5V), longer collarettes, and oblong conidia (Figs 5U–X, 14C).

#### ﻿ Neobelonopsis didymospora

Taxon classificationFungiHelotialesMollisiaceae

Itagaki & Hosoya sp. nov.

8955027C-B3DC-5A4E-ADD1-978A63F0D22B

MB842631

Figs 6 , 13 , 14D

##### Etymology.

Named after two-celled ascospores.

##### Diagnosis.

Resembles *Neobelonopsisbicolor*, but distinguishable by sparse, minute guttles in living/dead ascospores, shorter asci, and wider host range including woody bamboos.

##### Holotype.

TNS-F-13501, Yakushima Island, Kagoshima Pref., Japan, 19 October 2005, on decaying culms of woody bamboos, ex-holotype culture NBRC 115354.

##### Description.

***Apothecia*** superficial, without subiculum and scuta, 0.1–0.2 mm high, with blackish green (C100M100Y80–100K30) to black receptacle; disc 0.5–1 mm diam., white to bluish gray (C60M30–40Y20K60) when fresh, shrunk to 0.3–0.7 mm diam., cream (Y20K10) or olive (C40M40Y60–100K10) when dried. Ectal excipulum 30–50 µm thick at base, 20–25 µm thick at the upper flank to margin; cortical cells obovoid to clavate, (10–)12–15 × 7.5–10 µm at base, becoming slender and closely packed at the upper flank to margin, containing refractive vacuoles at the protruding cells when mounted fresh in water. Medullary excipulum 25–38 µm thick, frequently dichotomously branched, radially spreading toward the upper flask. ***Asci*** (50–)52–60(–65) × 5–7.5 µm, arising from croziers, with MLZ + apical pore. ***Ascospores*** 10–14(–16) × 2.5–3.5 µm, ellipsoid to fusiform, with subacute to acute extremes, frequently constrict at the septum, (0–)1–2-septate, hyaline, containing scattered small guttules. ***Paraphyses*** (45–)53–65 × 2.5–3(–4) µm, simple, (1–)2–3-septate, containing long refractive vacuoles in the apical cells and first 2–3 lower cells. ***Colony*** of NBRC 115354 on PDA flat, entire, dense, cottony to felted, dark brown (C60M80Y80–100K10) to beige (C10M20Y20–40K10) at the center, becoming pale brown toward to the edge, same colors at the reverse side, without soluble pigment and crystals; aerial mycelium sparse to dense, white to beige. ***Conidiophores*** solitary to occasionally aggregated on aerial hyphae, semi-macronematous, short, arising vertically or laterally from hyphae, pale to dark brown, smooth, thick-walled, sometimes branched 2–3 times, constricted at the septa, 2–3 µm width; ***phialides*** ampulliform, up to 15 µm long, 3.5 µm width at base, discrete or integrated, terminal or intercalary, hyaline to pale brown, thick-walled, with cylindrical to wide funnel-shape collarettes of 4.5–7.5 × 3 µm; ***conidia*** aseptate, subspherical to ellipsoid, abundantly aggregated in slimy head, 1.5–1.8 µm diam., hyaline, thin-walled.

**Figure 6.**
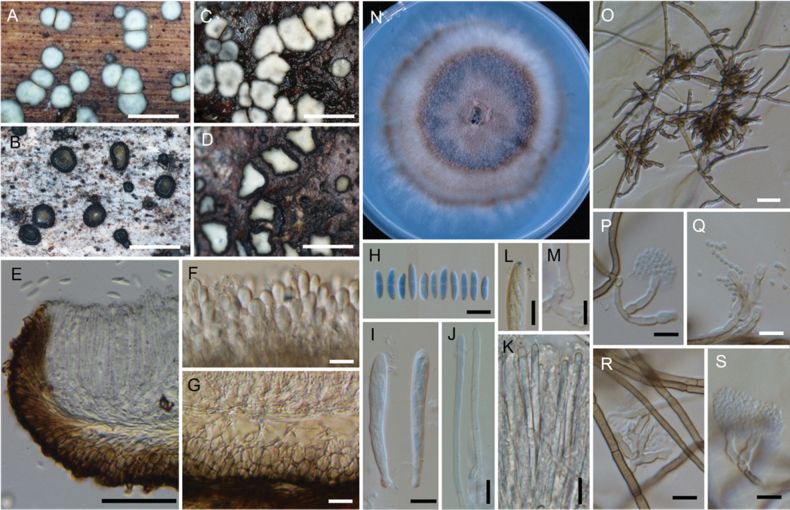
*Neobelonopsisdidymospora* (TNS-F-13501, holotype) **A** fresh apothecia on decaying Bamboo culm **B** dried apothecia on decaying Bamboo culm **C** fresh apothecia on decaying unidentified wood (TNS-F-86670) **D** dried apothecia on decaying unidentified wood (TNS-F-86670) **E** vertical section of the apothecium (in LA) **F** reflective vacuoles in fresh marginal cells (in water) **G** vertical section at the basal apothecium (in lactic acid) **H** ascospores (in CB/LA) **I** asci with ascospores (in CB/LA) **J** paraphyses (in CB/LA) **K** fresh paraphyses with long refractive vacuoles (in water) **L** blue-stained apical pore of ascus (in Melzer’s solution after 3% KOH pretreatment) **M** crozier at the base of ascus (in CB/LA) **N** three months old colony on PDA**O** clusters of conidiophores (in CB/LA) **P–S** conidiophores with conidia (in CB/LA). Scale bars: 1 mm (**A–D**); 50 μm (**E**); 20 μm (**O**), 10 μm (**F–M, P–S**).

##### Additional specimens examined.

TNS-F-13509, Yakushima Island, Kagoshima Pref., 19 October 2005, on decaying wood of *Elaeocarpusjaponicus*, culture NBRC 115651; TNS-F-86178, Shishizuka Pond, Tsuchiura City, Ibaraki Pref., 29 October 2018, on decaying wood of *Albiziajulibrissin*, culture NBRC 115657; TNS-F-88720, Shirokanedai, Meguro Ward, Tokyo, 6 July 2018, on dead stem of *Trachycarpusfortunei*; TNS-F-86661 and TNS-F-86652, Yugashima, Izu City, Shizuoka Pref., 15 October 2021 on decaying culms of woody bamboos and unidentified wood, respectively; TNS-F-86670, Kawazu City, Kamo County, Shizuoka Pref., 16 October 2021, on unidentified decaying wood; TNS-F-86718, Mt. Katsuu, Nago City, Okinawa Pref., 27 October 2021, on decaying wood of *Alnus* sp.

##### Notes.

*Neobelonopsisdidymospora* forms apothecia in autumn (October–December) and has a wide host range, but limited to woody plants, including woody bamboo. *Neobelonopsisdidymospora* forms its asexual stage only on CMA (Fig. 6O, S). This fungus is superficially similar to *N.bicolor*, but differs in fewer guttules in the cytoplasm.

Based on a BLAST search of the GenBank database, the closest hits to the ITS sequences of *N.didymospora* were three sequences of *Mollisia* sp. from New Zealand collected from the dead frond of *Rhopalostylissapida* [MG195516; Identities=553/554 (99.8%), no gaps], fallen unidentified wood [MG195517; Identities=551/554 (99.5%), one gap], and fallen wood of *Coriariaarborea* [MG195518; Identities=511/511 (100%), no gaps]. The presence of these sequence data suggests that distribution of *N.didymospora* is not limited in Japan, but also in New Zealand.

#### ﻿ Neobelonopsis microspora

Taxon classificationFungiHelotialesMollisiaceae

Itagaki & Hosoya sp. nov.

2A0FEADA-93AA-5E4F-9E4C-5F1843633BFD

MB842632

Figs 7 , 13 , 14E

##### Etymology.

Named after its small ascospores.

##### Diagnosis.

Characterized by narrow, aseptate ascospores.

##### Holotype.

TNS-F-18068, Yuzawa Town, Minami-uonuma County, Niigata Pref., Japan, 18 July 2006, on decaying culms of *Sasa* sp., ex-holotype culture NBRC 115567.

##### Description.

***Apothecia*** developed from scuta. ***Scuta*** superficial, scattered to gregarious, flat discoid, dark brown (C60M80Y80–100K60) to black, 125–450 µm diam., ***textura epidermoidea***. ***Apothecia*** 0.1–0.2 mm high, with grayish brown (C10–30M30–40Y60K60) receptacle; disc 0.3–1.5 mm diam., cream (Y10–30K10) when dried. Ectal excipulum 25–35 µm thick at base, 15–25 µm thick at the upper flask to margin; cortical cells hemispherical to obpyriform, 12–15(–17) × 7.5–11 µm at base, becoming smaller and hyaline at the upper flask to margin. Medullary excipulum, 25–50 µm thick. ***Asci*** (40–)45–55(–63) × 3.7–5 µm, arising from croziers, with MLZ + apical pore. ***Ascospores*** (7.5–)9.5–12.5(–16) × 2–2.5 µm, cylindrical to subcylindrical-fusoid-clavate with rounded extremes, aseptate, without guttules. ***Paraphyses*** (47–)52–62(–67.5) × 2–3.5(–4) µm, simple, rarely branched, (1–)2–3-septate. ***Subiculum*** thinly developed at the surface of substrates, sparse overall, shiny brown; subicular hyphae straight to undulate, frequently forming monilioid cells at the tip of the hyphae, 3–5 µm diam. with 0.5–1 µm thick-walls, perpendicularly branched. ***Colony*** of NBRC 115567 on PDA entire, flat to winkled at the center, floccose to felted, gray (K50–70) from the surface, darker from the reverse, without soluble pigment and crystals; aerial mycelium sparse to dense, white to pale gray. ***Conidiophores*** solitary to occasionally aggregated, semi-macronematous, short, arising vertically or laterally from fascicular hyphae, pale to dark brown, smooth, thick-walled, sometimes branched, constricted at the septa, 2.5–3 µm width; ***phialides*** ampulliform, up to 15 µm long, 3–4 µm width at base, discrete or integrated, terminal or intercalary, thick-walled, with cylindrical to long funnel-shape collarettes; collarettes of 6–8 × 2–3 µm, dark brown, occasionally covered with granules; ***conidia*** aseptate, cylindrical oblong to fusiform, abundantly aggregated in slimy heads, 4–5 × 1–1.5 µm, hyaline, thin-walled.

**Figure 7.**
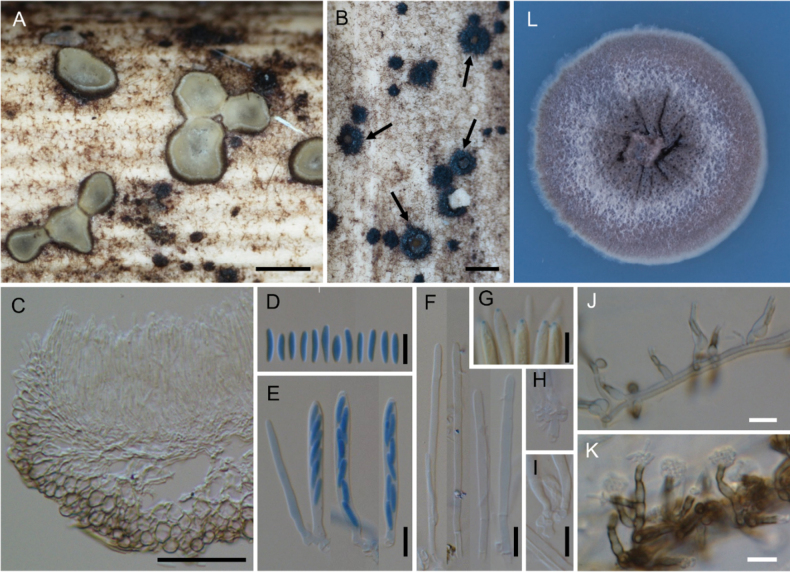
*Neobelonopsismicrospora* (TNS-F-18068, holotype) **A** dried apothecia on the decaying culm of *Sasa* sp. **B** immature apothecia protruding from the stromata (arrows) **C** vertical section of the apothecium (in LA) **D** ascospores (in CB/LA) **E** asci with ascospores (in CB/LA) **F** paraphyses (in CB/LA) **G** blue-stained apical pore of asci (in Melzer’s solution after 3% KOH pretreatment) **H, I** croziers at the base of asci (in CB/LA) **J** conidiophores with pigmented collarets **K** conidiophores. Scale bars: 0.5 mm (**A, B**); 50 μm (**C**); 10 μm (**D–K**).

##### Additional specimens examined.

TNS-F-16804, Sugadaira Montane Research Center, Ueda City, Nagano Pref., 7 July 2007, on unidentified fallen branches, culture NBRC 115653; TNS-F-17105, Nozori Lake, Kuni Village, Agatsuma County, Gunma Pref., 15 May 2004, on decaying culms of *Sasa* sp., culture NBRC 115650; TNS-F-86453, Shiromine, Shiroyama City, Ishikawa Pref., 18 June 2021, on decaying culms of *Sasapalmata*, culture NBRC 115660; TNS-F-86584, Kawakami Town, Noboribetsu City, Hokkaido, 2 August 2021, on decaying culms of *Sasakurilensis*, culture NBRC 115662.

##### Notes.

The minimum length of the ascospores of *N.microspora* is the shortest in *Neobelonopsis* but its maximum length is overlapped with the other species. This fungus resembles *B.eriophori* Raitv. in macroscopic appearance of apothecia and in having short, aseptate ascospores (16–19 × 3–3.5 µm), but ascospores of *B.eriophori* become uniseptate at maturity while that of *N.microspora* remain aseptate (Raitviir 2003),

*Neobelonopsismicrospora* produces conidiophores only on CMA, and the conidia germinate frequently (Figs 7J, K, 14E). The asexual morphology of *N.microspora* is very similar to that of *N.cinnabarina*, with long collarets and oblong conidia, except conidiophores do not form a sporodochium.

#### ﻿ Neobelonopsis multiguttata

Taxon classificationFungiHelotialesMollisiaceae

Itagaki & Hosoya sp. nov.

685E05E9-45D0-55BF-8AA5-6A7EEF95BD73

MB842635

Figs 8 , 13 , 14F

##### Etymology.

Named after the abundant number of guttules in the ascospores.

##### Diagnosis.

Resembles *N.acutata*, but distinguishable by more sparsely formed conidiophores, longer asci, and longer ascospores with rounded extremes (vs. more acute in *N.acutata*).

##### Holotype.

TNS-F-86402, Sugadaira Research Station, Mountain Science Center, Ueda City, Nagano Pref., 5 June 2021, on decaying culms of *Sasakurilensis*, ex-holotype culture NBRC 115371.

##### Description.

***Apothecia*** developed from scuta. ***Scuta*** superficial, scattered to gregarious, flat discoid, approximately 0.2 mm diam., blackish brown (C80M100Y80–100K60), ***textura epidermoidea***, consisting of thick-walled cells. ***Apothecia*** 0.2–0.4 mm high, with dark brown (C80M80Y80–100K60) receptacle; disc 0.5–1.6 mm diam., white to pale gray (K10) when fresh, shrunk to 0.4–1.3 mm diam., pale yellow (Y10) when dried. Ectal excipulum 37–50 µm thick at base, 25–35 µm thick at the upper flank to margin; cortical cells hemispherical to short clavate, 12–17 × 9–10(–12) µm at base, becoming slender and closely packed toward the upper flank to margin. Medullary excipulum 37–87 µm thick. ***Asci*** (63–)78–98(–105) × 5–8 µm, arising from croziers, with MLZ + apical pore. ***Ascospores*** (12–)17–26(–27.5) × 2.5–3.5 µm, long ellipsoid to fusiform with rounded extremes, (1–)3-septate, containing abundant guttules. ***Paraphyses*** (62–)74–90(–100) × 2.5–3 µm, simple, with long apical cell. ***Subiculum*** sparsely developed, covering the surface of substrates in patches, shiny brown; subicular hyphae straight to curved, usually constricted at septum, fascicular, 3–5 µm width with 0.5–1 µm thick-walls, septate every 15–25(–50) µm, branched at right angle, covered by gelatinous substance. ***Colony*** of NBRC 115371 on PDA flat to slightly winkled, entire to undulate, floccose to woolly, grayish brown (C20–30M40Y40K60) from the surface, forming indistinct section observed clearer from the reverse, without soluble pigment and crystals; aerial mycelium moderately abundant at the center, sparse at the edge, pale gray (K10–30) to white. ***Conidiophores*** semi-macronematous, short, arising vertically from aerial hyphae, pale to dark brown, smooth, thick-walled, constricted, occasionally loosely branched; ***phialides*** cylindrical to ampulliform, up to 16 µm long, 3 µm width at base, discrete, arranged terminal or intercalary, pale brown, thick-walled, with cylindrical funnel-shape collarettes of 4.5–6.5 × 2–3 µm; ***conidia*** aseptate, spherical to subspherical, abundantly aggregated in slimy heads, 1.5–1.7 µm diam., hyaline, thin-walled.

##### Additional specimens examined.

TNS-F-18023, Shirakamisanchi, Aomori Pref., 24 May 2006, on decaying culms of *Sasa* sp.; TNS-F-39229, Mt. Tsukuba, Tsukuba City, Ibaraki Pref., 22 April 2011, on decaying culms of *Sasa* sp.; TNS-F-54941, Omama Town, Midori City, Gunma Pref., 9 May 2018, on decaying culms of *Sasa* sp.; TNS-F-61278, Mt. Tsukuba, Tsukuba City, Ibaraki Pref., 16 April 2014, on fallen cupules of *Faguscrenata*; TNS-F-61280, Hakone Town, Ashigara-shimo County, Kanagawa Pref., 23 May 2014, on fallen cupules of *F.crenata* Blume, culture NBRC 115655; TNS-F-81133, Sugadaira Research Station, Montane Science Center, Ueda City, Nagano Pref., June 2017, on decaying culms of *Sasakurilensis*; TNS-F-86224, Sekimoto Town, Kita-ibaraki City, Ibaraki Pref., 3 June 2020, on dead branches on living *Stephanandraincisa*, culture NBRC 115365; TNS-F-86426, Mt. Amari, Asahi Town, Nirasaki City, Yamanashi Pref., 14 June 2021, on decaying culms of *Sasa* sp.; TNS-F-86465, Nagataki Town, Noumi City, Ishikawa Pref., 18 June 2021, on decaying culms of *Sasapalmata*, culture NBRC 115661.

**Figure 8.**
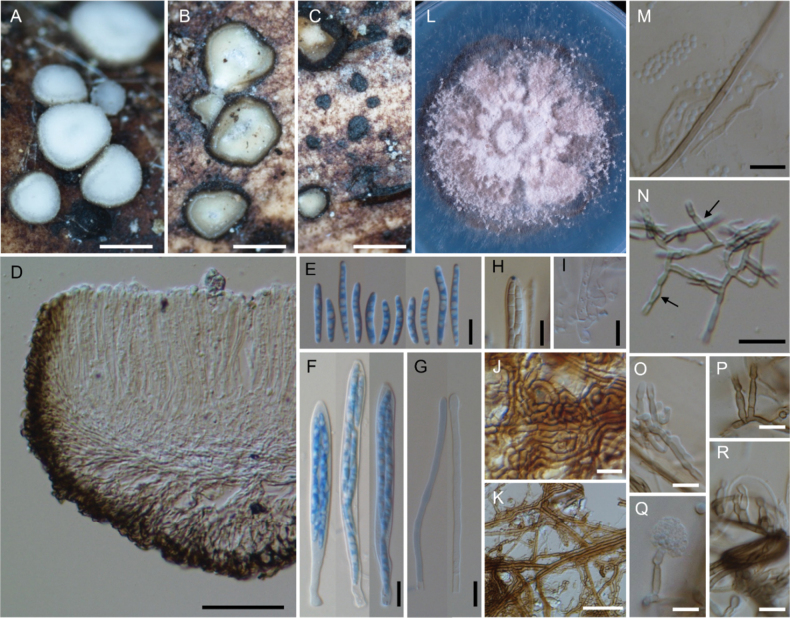
*Neobelonopsismultiguttata* (TNS-F-86402, holotype) **A** fresh apothecia on decaying culm of *Sasakurilensis***B** dried apothecia **C** stromata with sparse subiculum **D** vertical section of apothecium (in LA) **E** ascospores (in CB/LA) **F** asci with ascospores (in CB/LA) **G** paraphyses (in CB/LA) **H** blue-stained apical pore of ascus (in Melzer’s solution after 3% KOH pretreatment) **I** croziers at the base of asci (in CB/LA) **J** texture of stroma (in LA) **K** subicular hyphae (in lactic acid) **L** three months old colony on PDA**M** conidia (in CB/LA) **N–R** conidiophores, arrows in **N** indicated conidiogenous cells (in CB/LA). Scale bars: 0.5 mm (**A–C**); 50 μm (**D, K**); 20 μm (**N**), 10 μm (**E–J, M, O–R**).

##### Notes.

*Neobelonopsismultiguttata* has a wide host range, such as *Sasa* spp., *Faguscrenata*, and *Stephanandraincisa*, and occurs on various substrates, such as culms, branches, and cupules. *Neobelonopsismultiguttata* was found in spring and its morphology overlaps with *N.bicolor* in the dimensions of apothecia and paraphyses. However, the ITS sequence similarity with *N.acutata* is only 93.8%. Further, the two species form phylogenetically distinct clades (Fig. 1). The conidiophores of *N.multiguttata* on CMA are discrete (Figs 8N–R, 14F), rather than aggregated as in *N.acutata* (Fig. 3N, O).

Based on a BLAST search for the ITS sequences of *Neobelonopsismultiguttata* in the GenBank database, the closest hit was *Ascomycota* sp. (MK842071), isolated from the needles and roots of pine trees in South Korea [Identities=531/531 (100%), no gaps]. The endophytic isolate was recognized as *Mollisia* sp. by Rim et al. (2021). This result suggests that *N.multiguttata* has an endophytic phase as part of its life cycle.

#### ﻿ Neobelonopsis obtusa

Taxon classificationFungiHelotialesMollisiaceae

Itagaki & Hosoya sp. nov.

3427682B-4FA8-5BF9-B950-67825F08C12E

MB842637

Figs 9 , 13 , 14G

##### Etymology.

Named after rounded apices of ascospores.

##### Diagnosis.

Differs from *N.acutata* and *N.multiguttata*, which share 3-septate ascospores, by shorter ascospores with obtuse extremes and occurring only on woody substrates.

##### Holotype.

TNS-F-15602, Iryuda, Odawara City, Kanagawa Pref., 12 April 2007, on decaying wood of AucubajaponicaThunb.var.japonica, ex-holotype culture NBRC 115381.

##### Description.

***Apothecia*** superficial without subiculum and scuta, 0.2–0.3 mm high, with blackish brown (C80M80–100Y80–100K60) receptacle; disc 0.5–1.5 mm diam., white to pale gray when fresh, often turned grayish blue (C30–40M10Y10K30 or C40M20Y20K30) when moist, shrunk to 0.3–1 mm diam., pale yellow (Y20–30) or buff (M10Y30–40) when dried. Ectal excipulum 37–63 µm thick at base, 25–35 µm thick at the upper flank to margin; cortical cells hemispherical to short clavate, (10–)12–18 × (7–)8–12 µm at base, becoming slender and closely packed toward the upper flank to margin, containing refractive vacuoles at the protruding cells when mounted fresh in water. Medullary excipulum 60–75 µm thick, frequently dichotomously branched toward the margin. ***Asci*** (52–)56–78(–98) × 6–8.5(–10) µm, arising from croziers, with MLZ + apical pore. ***Ascospores*** (8–)13–17(–20) × 2.5–3.5 µm, subcylindrical with obtuse to subacute extremes, (1–)3-septate, containing small guttules. ***Paraphyses*** (40–)47–63(–70) × 2.5–3 µm, simple, (1–)2–3-septate, containing long refractive vacuoles at the apical cells when mounted fresh in water. ***Colony*** of NBRC 115381 on PDA entire, convex with abundant aerial hyphae, woolly to hairy, dark beige (M10Y20K30) from the surface, forming indistinct section and zonation observed clearer from the reverse, without soluble pigment and crystals; aerial mycelium abundant, membranous in the center, becoming densely fascicular, beige (C10–20M30Y30K10) to white. ***Conidiophores*** aggregated in inconspicuous clusters on aerial hyphae, (semi-)macronematous, constricted, arising vertically or laterally from hyphae, pale to dark brown, smooth, thick-walled, frequently branched; ***phialides*** ampulliform to lageniform with determinate collarettes, up to 15 µm long, approximately 3 µm width at base, discrete to integrated, terminal or intercalary, pale brown, thick-walled, with cylindrical to wide funnel-shape collarettes of 4–6.5 × 2–3 µm; ***conidia*** aseptate, spherical to subspherical, abundantly aggregated in slimy heads, 2–2.5 µm diam., hyaline, thin-walled.

**Figure 9.**
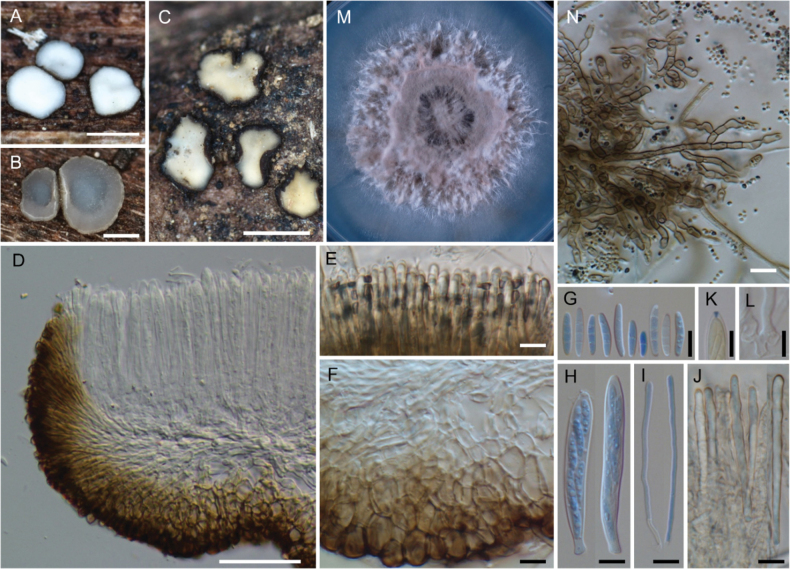
*Neobelonopsisobtusa* (TNS-F-15602, holotype) **A** fresh apothecia on decaying unidentified wood **B** fresh apothecia during moist conditions **C** dried apothecia **D** vertical section of the apothecium (in LA) **E** refractive vacuoles in fresh marginal cells (in water) **F** vertical section at the basal apothecium (in LA) **G** ascospores (in CB/LA) **H** asci with ascospores (in CB/LA) **I** paraphyses (in CB/LA) **J** fresh paraphyses with long refractive vacuoles (in water) **K** blue-stained apical pore of ascus (in Melzer’s solution after 3% KOH pretreatment) **L** croziers at the base of asci (in CB/LA) **M** three months old colony on PDA**N** conidiophores with conidia (in water). Scale bars: 1 mm (**A, C**); 0.5 mm (**B**); 50 μm (**D**); 10 μm (**E–L, N**).

##### Additional specimens examined.

TNS-F-44017, Yoyogi, Shibuya Ward, Tokyo, 8 November 2011, on unidentified decaying wood, culture NBRC 115654; TNS-F-54934, Omama Town, Midori City, Gunma Pref., 21 April 2018, on unidentified decaying wood, culture NBRC 115656; TNS-F-86359, Mt. Yamizo, Daigo City, Kuji County, Ibaraki Pref., 24 May 2021, on decaying wood of *Lindera* sp., culture NBRC 115659; TNS-F-86638, Ikaho, Shibukawa Town, Gunma Pref., 5 October, 2021, on decaying wood of *Quercus* sp.; TNS-F-86658, Yugashima, Izu City, Shizuoka Pref., 15 October 2021, on decaying wood of *Cornuscontroversa*, culture NBRC 115664; TNS-F-86668, Kawazu City, Kamo County, Shizuoka Pref., 15 October 2021, on decaying wood of *Morusaustralis*.

##### Notes.

The ectal excipulum consisting of closely packed brownish cells of *Neobelonopsisobtusa* is similar to that of *N.didymospora*. However, the two species can be easily distinguished by the stable number of septa of ascospores (3-septate vs. 1-septate). *Neobelonopsisobtusa* forms an asexual stage on CMA (Figs 9N, 14G) which closely resembles that of *N.acutata* in dendroid (irregularly branched) conidiophores (Figs 3N–O, 14A).

#### ﻿ Neobelonopsis ramosa

Taxon classificationFungiHelotialesMollisiaceae

Itagaki & Hosoya sp. nov.

DB6C0BBC-91AF-5124-9BA2-767B6EEF5D5F

MB842634

Figs 10 , 13

##### Etymology.

Named after the frequently branched paraphyses.

##### Diagnosis.

Characterized by multi-septate, frequently 1–3 times branched paraphyses and long ascospore with 0–3 septum.

##### Holotype.

TNS-F-86030, Daimyoujin Fall, Ueda City, Nagano Pref., 6 August 2018, on decaying culms of *Sasa* sp., ex-holotype culture NBRC 115362.

##### Description.

***Apothecia*** developed from scuta. ***Scuta*** superficial, scattered to gregarious, flat discoid, 140–185 mm diam., dark brown (C40–60M80Y80K60), consisting of closely packed brown cells and hyphae with thick-walls. ***Apothecia*** 0.1–0.2 mm high, with dark brown (C60M80Y80–100K60) receptacle; disc 0.1–1.5 mm diam., cream (Y10–30K10) when dried. Ectal excipulum 37.5–45 µm thick at base, 25–37 µm thick at the upper flank to margin; cortical cells pyriform to short clavate, paler toward to margin, 11–14(–16) × 7–10 µm at base, becoming slender and smaller toward margin. Medullary excipulum 25–63 µm thick. ***Asci*** (63–)74–88(–98) × 5–7.5 µm, arising from croziers, with MLZ + apical pore. ***Ascospores*** (12–)16–22(–25) × 2.5–3 μm, long subcylindrical to fusiform, with subacute extremes, 0–3-septate, sparsely containing guttules. ***Paraphyses*** (60–)65–77(–85) × 2–2.5 µm, frequently branching 1–3 times at the middle cells, multi-septate. ***Subiculum*** covering the surface of substrates in patches, sparse to moderately abundant around the scuta and apothecia, shiny brown; subicular hyphae straight or gently curved, sometimes forming fascicules with 2–3 hyphae, approximately 5 µm diam. with 0.5–1 µm thick-walls, septate every 20–50 µm, perpendicularly branched, covered by gelatinous substance. ***Colony*** of NBRC 115362 on PDA undulate, flat, floccose to cottony, sepia (C30–60M100Y60–80K60) from the surface and near the center, paler toward the margin, forming an indistinct section, darker from the reverse, without soluble pigment and crystals; aerial mycelium sparse to moderately abundant at the center, white to beige.

**Figure 10.**
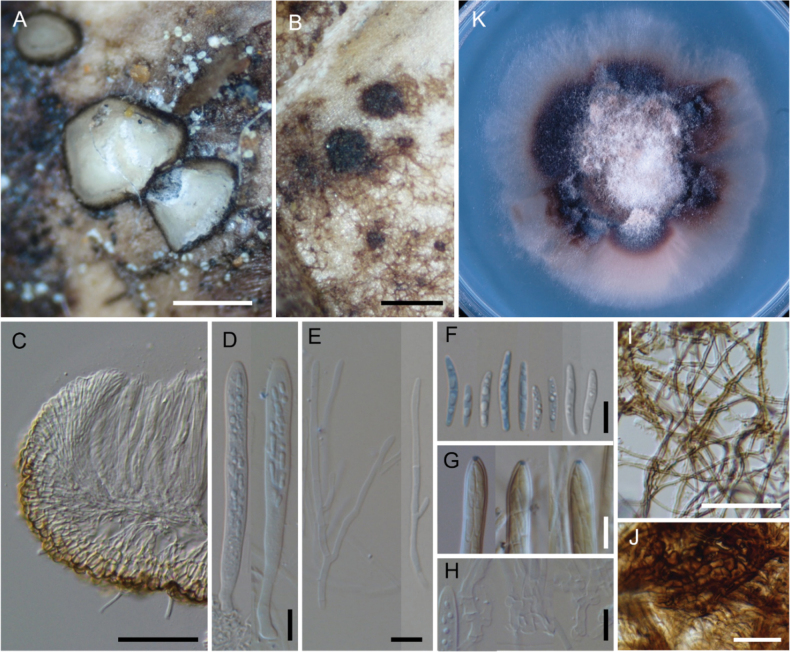
*Neobelonopsisramosa* (TNS-F-86030, holotype) **A** dried apothecia on the decaying culm of *Sasa* sp. **B** stromata with sparse subiculum **C** vertical section of the apothecium (in LA) **D** asci (in CB/LA) **E** paraphyses (in CB/LA) **F** ascospores (in CB/LA) **G** blue-stained apical pore of asci (in Melzer’s solution after 3% KOH pretreatment) **H** croziers at the base of asci (in CB/LA) **I** subicular hyphae (in LA) **J** texture of stroma (in LA) **K** three months old colony on PDA. Scale bars: 0.5 mm (**A**); 0.25 mm (**B**); 50 μm (**C, I**); 20 μm (**J**); 10 μm (**D–H**).

##### Notes.

*Neobelonopsisramosa* is morphologically distinguished from other *Neobelonopsis* species by its frequently branching paraphyses (Figs 10E, 12). *Neobelonopsismicrospora* also have branched paraphyses (mostly branched once), but differ from *N.ramosa* in ascospore with a stable number of septum (1- or 0-sepatate, respectively). *Belonopsispamparum* Speg. resembles *N.ramosa* in habitat (apothecia on culm of poaceous grass, *Aristida*) and having frequently branched paraphyses, but differs in having larger ascospores (30–35 × 3–4 μm) with 5–7-pseudosepta (Spegazzini 1909). No asexual stage was observed in colonies of NBRC 115362 on artificial media.

#### ﻿ Trichobelonium albobarbatum

Taxon classificationFungiHelotialesMollisiaceae

Itagaki & Hosoya sp. nov.

E66A465A-2E77-56B0-97F0-30A42DB799FA

MB842638

Figs 11 , 13 , 14H

##### Etymology.

Named after the anchoring hyphae between the cortical cells of receptacle and subiculum, which resembles a white beard (*albo* and *barbata* in Latin, respectively).

##### Diagnosis.

Resembles *T.kneiffii*, but distinguished by its larger ascospores.

##### Holotype.

TNS-F-86430, Sawara Pond, Asahi Town, Nirasaki City, Yamanashi Pref., 14 June 2021, on decaying poaceous grass culm lying on the wet ground close to the pond, ex-holotype culture NBRC 115568.

##### Description.

***Apothecia*** developed scuta. ***Scuta*** superficial, scattered to gregarious, flat to protruded discoid, 125–375 µm diam., blackish brown (C80M100Y80–100K60), ***textura epidermoidea***. ***Apothecia*** sessile, globose to pulvinate when immature, discoid to saucer-shape when mature, flat to concave when fresh, doliiform to pulvinate when dried, 0.1–0.3 mm high, with brown (C40–80M80Y100K30) receptacle; disc 0.5–1.5 mm diam., entire to undulate, without hairs at margin, waxy, yellow (Y30–60) when fresh, shrunk to 0.2–1 mm diam., pulverulent, yellowish orange (M10–40Y80–100) when dried, turned to brown (C30–60M80Y80–100) with senescence. Ectal excipulum 30–40 µm thick at base, 20–30 µm thick at the upper flank to margin, ***textura globulosa*** and ***angularis***, composed of 2–4 layers of brown thick-walled cells; cortical cells hemispherical, 10–15(–17) × 6–10(–12) µm, ending up in cylindrical clavate cells, thick-walled, paler toward the margin; anchoring hyphae connecting the cortical cells of the flank and subiculum, radially extending from apothecium, 2.5–3 µm width, septate every 20–35 µm, thin-walled, hyaline, becoming conspicuous when apothecia dried. Medullary excipulum 100–150 µm thick, ***textura intricata*** to ***prismatica***, hyaline, containing crystals below giving a rough texture, composed of loosely interwoven thin-walled hyphae which is frequently dichotomously branching. ***Asci*** (75–)85–100(–107) × 12–16(–20) µm, cylindrical-clavate to saccate, 8-spored, arising from croziers, containing yellowish oil globules in cytoplasm that disappear when mature, with a thick-walled conical apex; apex MLZ+ with or without 3% KOH pretreatment. ***Ascospores*** (25–)30–35(–38) × 4.5–6 µm, fusiform-clavate, with rounded or subacute extremes, straight to sigmoid curved, thin-walled, (0–)3-septate, sometimes constricted at the septum, hyaline, with numerous guttules. ***Paraphyses*** 85–100(–115) × 2.5–4.5 µm, occasionally branching at base, cylindrical, often becoming slightly wider toward the apex, 2–3-septate, thin-walled, hyaline, containing fragmented refractive vacuoles when mounted fresh in water. ***Subiculum*** covering the surface of substrates in patches, sparse to especially abundant around the apothecia and scuta, shiny dark brown, consisting of 1–3 layers of closely packed subicular hyphae; subicular hyphae 2–5 µm diam., thick-walled, brown. ***Colony*** of NBRC 115568 on PDA entire to partially filamentous at the margin, flat to slightly convex with aerial hyphae, cottony to woolly, agate (C10–30M60Y60) to amber (C10–40M100Y60K60) from the surface, appearing maroon (C10–40M100Y60K60) from reverse, with apricot (M20–40Y60) soluble pigment uniformly diffuse in agar; crystals aggregating plate-like or small clusters, acicular, moderately abundant on colony surface and surrounding agar, 0.1–0.3 mm across, pale yellow (Y10–20); aerial mycelium especially abundant in the center and edge, blush pink (M20–30Y20); mycelium containing guttules, pale to dark brown, thick-walled, sometimes covered with exudates. ***Conidiophores*** (semi-)macronematous, arising vertically or laterally from hyphae, pale to dark brown, smooth, containing oil globules in the hyphal cell, constricted at the septum, thick-walled, occasionally 2–3 series of branches, 2–3 µm width; ***phialides*** cylindrical to ampulliform, up to 10 µm long, 2.5–4 µm width, discrete to integrated, terminal, pale brown, thick-walled, with short cylindrical or wide funnel-shape collarettes of 2.5–4 × 3 µm at the upper edge, hyaline to pale brown, thin-walled; conidia aseptate, ellipsoid, abundantly aggregated near the collarettes, 2–3 × 1 µm, hyaline, thin-walled.

**Figure 11.**
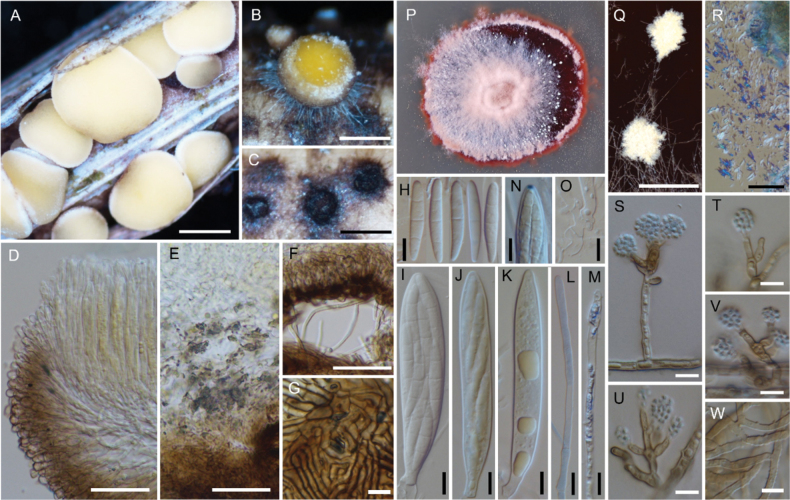
*Trichobeloniumalbobarbatum* (TNS-F-86430, holotype) **A** fresh apothecia on the decaying culm of unidentified grass (Poaceae) **B** dried apothecium with hyaline connective hyphae **C** protruding immature apothecia from the stromata **D** vertical section of the apothecium (in LA) **E** crystals in medullary excipulum (in LA) **F** connecting hyphae between ectal excipulum and subiculum (in LA) **G** texture of the stroma (in LA) **H** ascospores (in CB/LA) **I** ascus with mature ascospores (in CB/LA) **J** ascus with immature ascospores (in CB/LA) **K** immature ascus containing yellowish oil globes in the cytoplasm (in CB/LA) **L** paraphysis (in CB/LA) **M** rehydrated paraphysis with fragmented refractive vacuoles (in water) **N** blue-stained apical pore of ascus (in Melzer’s solution after 3% KOH pretreatment) **O** croziers at the base of asci (in CB/LA) **P** three months old colony on PDA**Q** yellow crystals formed on hyphae **R** acicular crystals (in water) **S–V** conidiophores with conidia (in CB/LA); W hyphae with exudates (in CB/LA). Scale bars: 0.5 mm (**A, Q**); 0.25 mm (B, C); 50 μm (**D–F**); 20 μm (**R**); 10 μm (**G, H–O, S–W**).

##### Notes.

The yellowish color of the hymenium is due to the oil globules in immature asci (Fig. 11D, K). The oil globules gradually disappear as the ascospores mature (Fig.) . *Trichobeloniumalbobarbatum* forms conidiophores immersed in agar, especially at the bottom of the Petri dish (Figs 11S–V, 14H). Both *T.albobarbatum* and *T.kneiffii* have well-developed dark brown subiculum, white anchoring hyphae, yellow hymenium, abundant crystals in excipulum, long asci (approximately 80–100 µm length), and 1–3-septate ascospores (Schröter 1908). However, *T.albobarbatum* has wider asci (vs. 5–6 µm width) and larger ascospores (vs. 16–18 × 2–2.5 µm) than *T.kneiffii*.

#### ﻿ Trichobelonium miscanthi

Taxon classificationFungiHelotialesMollisiaceae

Itagaki & Hosoya sp. nov.

77BC3F0E-25D9-5CD3-974C-13953D4647A7

MB842639

Figs 12 , 13 , 14I

##### Etymology.

Named after the genus of its host, *Miscanthus*.

##### Diagnosis.

Characterized by 5-septate ascospores and sparse subiculum

##### Holotype.

TNS-F-17835, Sugadaira Montane Research Center, Ueda City, Nagano Pref., 17 September 2005, on decaying culm of *Miscanthussinensis*, ex-holotype culture NBRC 115566.

##### Description.

***Apothecia*** developed from scuta. ***Scuta*** superficial, scattered to gregarious, flat discoid, 145–180 µm diam., dark brown (C80M100Y80K60), ***textura epidermoidea***, gradually becoming ***textura porrecta*** and connecting to subiculum. ***Apothecia*** sessile, globose to pulvinate when immature, discoid to saucer-shape when mature, flat to slightly convex when fresh, 0.2 mm high, with brown (C30–60M60Y80–100K60) receptacle; disc 0.5–1.5 mm diam., entire to slightly undulate, without hairs at margin, waxy, white to pale yellow (Y10–30) when fresh, shrunk to 0.3–1 mm diam., pulverulent, cream (Y20–40K10) when dried. Ectal excipulum 25–35 µm thick at base, approximately 25 µm thick at the upper flank to margin, ***textura globulosa*** and ***angularis***, composed of 2–4 layers of brown thick-walled cells, not gelatinized, without crystals or exudates; cortical cells in middle to lower flank pyriform to clavate, with protruded cells, 16–21(–23) × 5–7 µm, containing with refractive vacuoles at margin when mounted fresh in water; anchoring hyphae connecting the cortical calls of the flank and subiculum, radially extending from apothecium, 2.5–3 µm width, thin-walled, brown. Medullary excipulum 40–75 µm thick, ***textura intricata*** to ***prismatica***, hyaline, composed of loosely interwoven thin-walled hyphae which is frequently dichotomously branching. ***Asci*** (77–)79–85(–90) × 12.5–15(–17.5) µm, cylindrical-clavate to saccate, 8-spored, arising from croziers, containing hyaline oil globules in cytoplasm that disappear when mature, with a thick-walled conical apex; apex MLZ+ with or without 3% KOH pretreatment. ***Ascospores*** (32–)37–47(–57.5) × 4.5–5.5 µm, long fusiform, with acute extremes, curved to sigmoid, occasionally constricted, thin-walled, often 5-septate, hyaline, containing large or abundant minute guttles. ***Paraphyses*** (70–)83–105(–115) × 2.5–3(–4) µm, simple, occasionally branching at base, cylindrical, often becoming slightly wider toward the apex, 2–3-septate, thin-walled, hyaline, (2–)3-septate, containing long refractive vacuoles when mounted fresh in water. ***Subiculum*** thinly developed the surface of substrates in patches, sparse to especially abundant around the mature apothecia, shiny brown; subicular hyphae straight to curved, usually swelling in a globose, 3–5 µm diam. with 0.5–1 µm thick-walls, septate every 15–30 µm, perpendicularly branched. ***Colony*** of NBRC 115566 on PDA, flat to slightly convex with aerial hyphae, cottony, grayish orange (C0–20M60Y60K10) from the surface, appearing cinnamon (C20–40M80Y100K30) from reverse side, with apricot (M20–40Y60) soluble pigment uniformly diffuse in agar; aerial mycelium dense, white to pale yellow (Y10–20); crystals aggregating plate-like or small clusters, acicular, moderately abundant on colony surface and surrounding agar, 0.1–0.5 mm across, pale yellow. ***Conidiophores*** macronematous to mononematous, arising from subicular hyphae, straight, pale to dark brown, thick-walled, smooth, 2–3 µm width; ***phialides*** ampulliform, up to 15 µm long, 2.5–4 µm width, integrated, arranged penicillately, pale brown, thick-walled, with cylindrical to wide funnel-shape collarettes of 3.5–5 × 2–3 µm; ***conidia*** aseptate, spherical to subspherical, 2–2.5 µm diam., hyaline, thin-walled.

**Figure 12.**
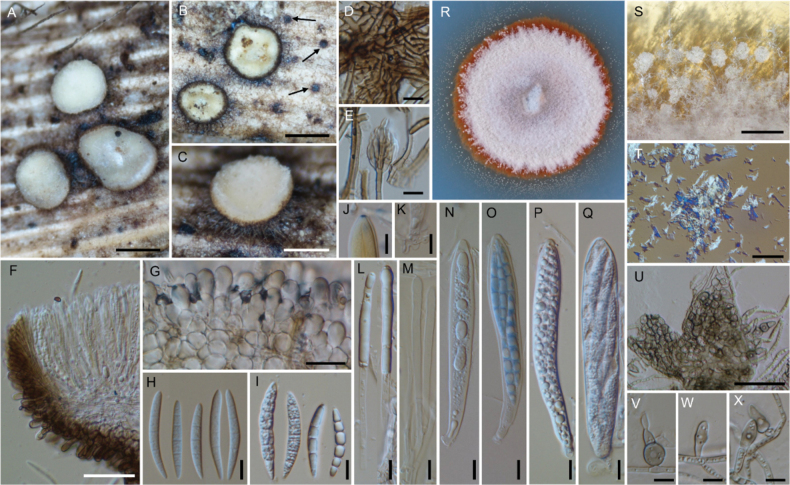
*Trichobeloniummiscanthi* (TNS-F-17835, holotype) **A** fresh apothecia on the decaying culm of *Miscanthussinensis***B** dried apothecia with stromata (arrows) **C** brown connective hyphae extending from the base of fresh apothecium **D** texture of stroma (in LA) **E** conidiophore (in LA) **F** vertical section of the apothecium (in LA) **G** fresh outermost cells of ectal excipulum with refractive vacuoles (in water) **H** ascospores (in CB/LA) **I** fresh ascospores with droplets (in water) **J** blue-stained apical pore of ascus (in Melzer’s solution after 3% KOH pretreatment) **K** croziers at the base of ascus (in CB/LA) **L** long refractive vacuoles in fresh paraphysis (in water) **M** branching paraphysis (in CB/LA) **N** immature ascus containing oil globes in the cytoplasm (in CB/LA) **O** ascus with mature ascospores (in CB/LA) **P** immature ascus (in water) **Q** ascus with ascospores (in water) **R** one month old colony on PDA**S** pale yellow crystals formed on the edge of the colony **T** acicular crystals (in water) **U** hyphal mass attached to the bottom of the Petri dish **V–X** swollen cells with melanized appressorium-like structure. Scale bars: 1 mm (**S**); 0.5 mm (**A, B**); 0.25 mm (**C**); 50 μm (**F, U**); 20 μm (**G, T**); 10 μm (**D, E, H–Q, V–X**).

##### Additional specimens examined.

TNS-F-30037, Hachimantai City, Iwate Pref., 12 October 2009 on decaying culm of *Miscanthussinensis*, culture NBRC 115652; TNS-F-81751, Kiritappu Wetland, Hamanaka City, Akkeshi County, Hokkaido, 29 August 2019, on decaying culm of *Phragmitesaustralis*; TNS-F-86581, Higashi Ward, Sapporo City, Hokkaido, 13 August 2021, on decaying culm of *Ph.australis*; TNS-F-86672 (culture NBRC 115667) and 86695, Yuzawa Town, Minami-uonuma County, Niigata Pref., 17 and 31 October 2021 (respectively), on decaying culm of *M.sinensis*; TNS-F-86700, Daigenta Lake, Yuzawa Town, Minami-uonuma County, Niigata Pref., 31 October 2021, on decaying culm of *M.sinensis*, culture NBRC 115668; TNS-F-86715, Toukamachi City, Niigata Pref., 31 October 2021, on decaying culm of *M.sinensis*.

##### Notes.

*Trichobeloniummiscanthi* occurs with *Neobelonopsiscinnabarina* as they share the same host, *Mollisiasinensis*, and fruiting season (autumn). Brown phialides (Fig. 12E) and spherical conidia, regarded as asexual stage of *T.miscanthi*, were observed to accompany subiculum, but we could not induce conidial reproduction under culture.

From the reverse of the two months old colony of *T.miscanthi* on CMA, clumps of dark cells strongly attached to the bottom of the Petri dish (Fig. 12U) were observed. The clumps are composed of swollen cells with melanized ring. The swollen cell is usually obovoid to pyriform, sometimes lobed or hyphoid, 10–15 × 6–10 µm, arising vertically from hyphae, thick-walled, and containing abundant guttles (Figs 12V–X, 14I). The brown ring structure has an outer diameter of 8–10 µm and inner diameter of 2–3 µm, and is formed at the cell and Petri dish interface. Very similar hyphal structures were reported by Aebi (1972) in the culture of *T.kneiffii*, but its function is unknown. The clumps of dark cells of *Phialocephalabamuru* P.T.W. Wong & C. Dong, known as plant pathogen, are interpreted as appressoria with infected pegs (Wong et al. 2015). Although this structure may be appressorium, direct observation of the mycelium of *T.miscanthi* on the host epidermis and inoculation experiments are needed to clarify whether the clamps of *T.miscanthi* function as an appressorium during the infection process.

**Figure 13.**
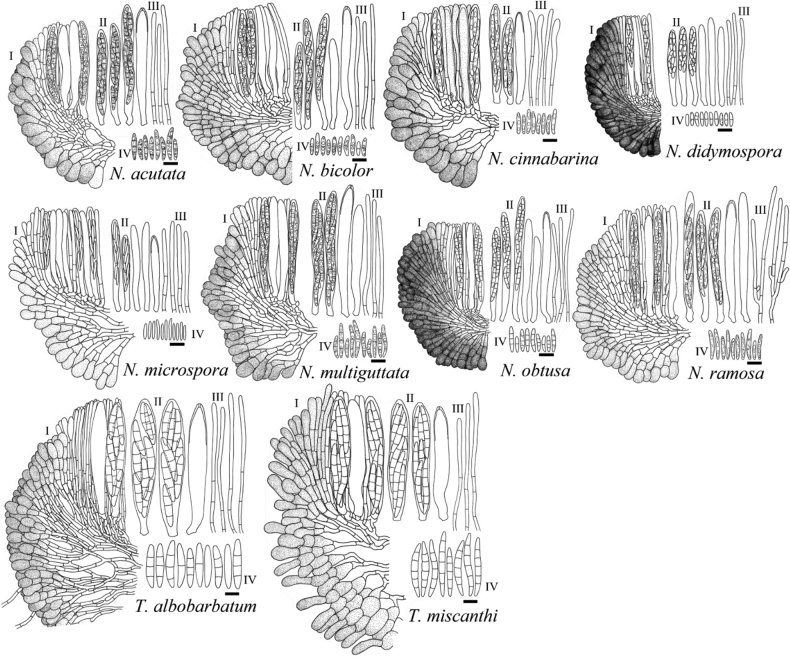
Line-drawings of the *Neobelonopsis* and *Trichobelonium* species **I** vertical section of the apothecium showing the marginal structure of ectal excipulum, medullary excipulum, and hymenium **II** asci **III** paraphyses **IV** ascospores. Scale bars: 10 μm.

*Trichobeloniummiscanthi* resembles *T.albobarbatum* in remarkable oil globules in immature asci, anchoring hyphae, and saccate form of asci. Although *T.miscanthi* lacks crystals in the excipulum, the culture produced abundant acicular crystals on PDA (Fig. 12R, S, T).

## ﻿Discussion

### ﻿Taxonomic treatment of *Mollisiadiesbachiana*

*Mollisiadiesbachiana* is morphologically characterized by narrow, cylindrical-oblong ascospores [(7–)7.5–8(–9) × 2 µm] (Tanney and Seifert 2020). Based on phylogenetic analysis (Fig. 1), *M.diesbachiana* is situated in the *Neobelonopsis* lineage. Although the morphology of *M.diesbachiana* is nearly identical to that of *Mollisiasensu stricto*, we proposed to transfer *M.diesbachiana* to *Neobelonopsis* to maintain monophyly of *Neobelonopsis*.

**Figure 14.**
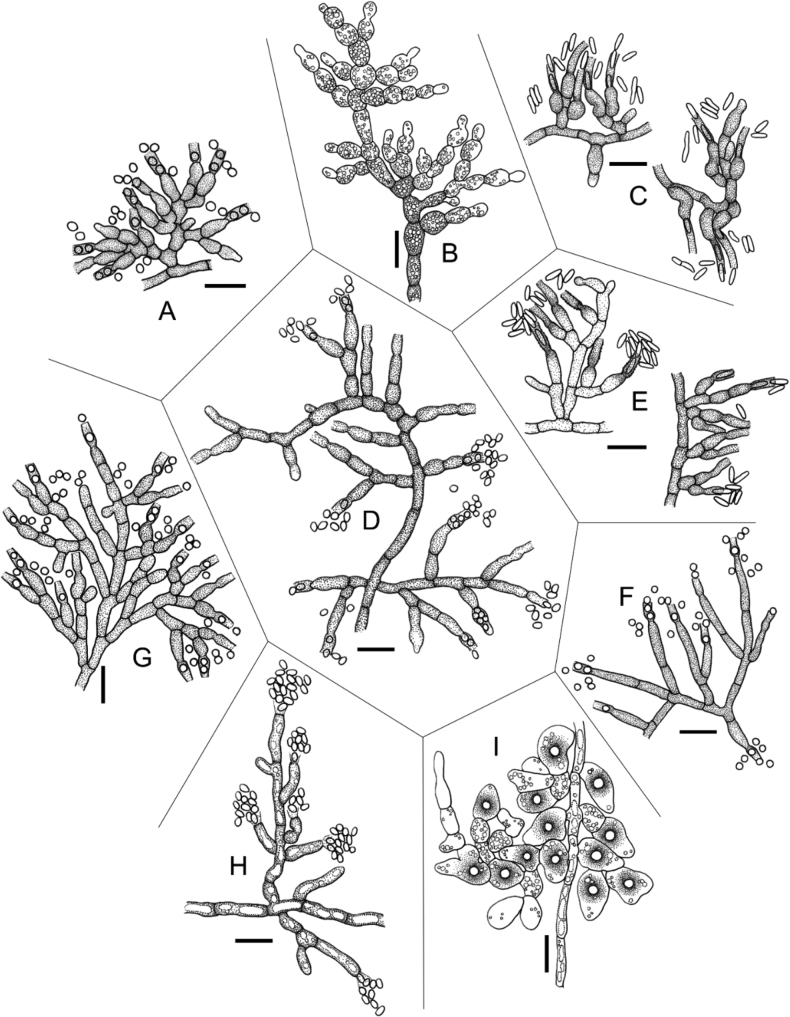
Asexual morph and hyphal structures of the *Neobelonopsis* and *Trichobelonium* species **A** conidiophores of *N.acutata***B** monilioid hyphae of *N.bicolor***C** conidiophores with conidia of *N.cinnabarina***D** conidiophores with conidia of *N.didymospora***E** conidiophores with conidia of *N.microspora***F** conidiophores with conidia of *N.multiguttata***G** conidiophores with conidia of *N.obtusa***H** conidiophores with conidia of *T.albobarbatum***I** hyphal mass with melanized appressorium-like structure of *T.miscanthi*. Scale bars: 10 μm.

#### ﻿ Neobelonopsis diesbachiana

Taxon classificationFungiHelotialesMollisiaceae

(Tanney & Seifert) Itagaki & Hosoya comb. nov.

D806BD39-D219-57F8-8648-F044DE1C12DA

MB846429

##### Basionym.

*Mollisiadiesbachiana* Tanney & Seifert, Studies in Mycology 95: 331, 2020.

### ﻿Taxonomic position of “*Belonopsis*” *excelsior*

Recently, “*Beloniumexcelsior*” has been adopted as the current name of this species in Index Fungorum. Nauta and Spooner (2000) characterized the genus *Belonium* by its excipulum with dark-walled globose material (grana), brown hair-like elements, or irregularly thick-walls. However, the description of *B.excelsior* by Rehm (1891) cited below did not match these characteristics: “Apothecien gesellig, sitzend, zuerst kuglig geschlossen, rundlich sich öffnend und die schüsselförmige, flache, zart berandete, hellfarbige Fruchtscheibe entblössend, aussen bräunlich, glatt, wachsartigweich” (apothecia gregarious, sessile, initially globose, opening roundly and becoming bowl-shape, with delicate margin, flat, light color at disc, brown at receptacle, smooth, waxy-soft, translated by ourselves). This species shares morphology with *Neobelonopsis*, such as white disc and blackish receptacle, but differs in habitat (submerged grasses culm), abundant crystals in excipulum, and extremely long (42–50 × 3–4 µm) ascospores with 7–10-septa (Rehm 1891, Ingold 1954, Nannfeldt 1985).

The phylogenetic tree inferred from ITS–LSU–RPB1 sequences (Fig. 1) obtained in this study placed “*Belonopsis*” *excelsior* (CBS 140.52) within a poorly supported clade containing *M.fusca* (CBS 555.63), *M.lividofusca* (CBS 231.71), and Mollisiacf.fusca (DAOMC 251565). In the ITS phylogenetic tree (Fig. 2), “*Belonopsis*” *excelsior* is at least included in Mollisiaceae, while this species is apart from *Cejpiahystrix* (=former type species of *Belonium*, *B.graminis*), which was contained neither in Mollisiaceae nor Pyrenopezizaceae. So, decisive taxonomic treatment of *B.excelsior* should wait until more appropriate analysis in Mollisiaceae lineage is given. To elucidate whether the existing *Belonopsis* species belong to *Neobelonopsis* or other mollisioid genera, further acquisition of sequence data and detailed morphological studies are needed.

### ﻿Justification to establish the new genus *Neobelonopsis*

Given the polyphyly of most genera in Mollisiaceae, Tanney and Seifert (2020) proposed three nomenclatural and taxonomic options for treating new taxa: 1) all taxa with diverse morphology and ecology are lumped together in a single genus, *Mollisia*; 2) accepting the non-monophyly of *Mollisia*, only morphologically divergent taxa are regarded as distinct genera; and 3) taxa are divided and assigned to genera erected or maintained based principally on monophyly.

As *Neobelonopsis* and *Mollisiasensu stricto* are strongly supported as a monophyletic clade (Fig. 1) together with other genera, a possible parsimonious proffer is to include all species of *Neobelonopsis* in the genus *Mollisia* in accordance with option 1. This proposal may avoid the construction of a vulnerable taxonomic system of Mollisiaceae characterized by many small and nomenclaturally unstable genera (Tanney and Seifert 2020). However, this approach extremely expands and obscures the generic concept of *Mollisia*. *Neobelonopsis* is acceptable not only by forming a well-supported monophyletic clade, but also morphologically differs from *Mollisiasensu stricto* featured by its longer ascospores.

As per option 2, Tanney and Seifert (2020) treated morphologically divergent lineages (such as *Loramyces*, *Obtectodiscus*, and *Ombrophila*) and ecologically remarkable lineages (such as *Phialocephala* and *Acephala* known as endophytes) as distinct genera. However, we did not follow option 2 because the criteria for recognizing a new lineage as a genus are not clearly defined.

Option 3 is the most acceptable taxonomic treatment, dividing Mollisiaceae into monophyletic genera through a polyphasic approach combining molecular phylogenetic analysis, morphology, and ecology (including host specificity and phenology of apothecial production). *Neobelonopsis* forms a phylogenetically well-supported clade and morphology that shows distinction from the traditional genus *Trichobelonium*.

### ﻿New characteristics of the genus *Trichobelonium*

*Trichobelonium* has been treated as a synonym of *Belonopsis* (Aebi 1972; Nauta and Spooner 1999), but multi-gene analysis revealed that *Trichobelonium* species newly described in this study are phylogenetically distinct from other genera in Mollisiaceae (Fig. 1). The morphology of *T.albobarbatum* and *T.miscanthi* is consistent with the original description of Beloniumsubgen.Trichobelonium Sacc. (Saccardo 1889). Detailed morphological observations elucidated the following new features of *Trichobelonium*: the presence of anchoring hyphae between the base of apothecium and the subiculum, the presence of oil globules in young asci disappearing as the ascospore maturity, and the production of abundant crystals and soluble pigments in the colonies. From these results, we propose to retain the genus, *Trichobelonium*. The type species, *T.kneiffii*, lacks DNA sequences, but its morphological features such as abundant crystals in excipulum, long ascospores with multi-septum, and well-developed subiculum indicate that it is congeneric with the two new species. The presence of the anchoring hyphae of *T.kneiffii* was also described as “filzigen oder spinnwebartigen Unterlage sitzend” (cobweb manner hyphal structure, translated by ourselves) by Schröter (1908).

### ﻿Trends in the morphological evolution of *Neobelonopsis* ascospores and conidia

In the *Neobelonopsis* clade (Fig. 1), the terminally positioned *N.acutata*, *N.obtusa*, and *N.multiguttata* have 3-septate ascospores, while *N.cinnabarina*, *N.bicolor*, *N.didymospora*, and *N.microspora* at the basal position have fewer (one) or no septa. *Neobelonopsisramosa* situated in mid-position has 0–3-septate ascospores, which might be an intermediate morphology between 0–1-septate and 3-septate species. Thus, ascospores were suggested to have more septa in *Neobelonopsis* in the terminal clades. Likewise, the phylogenetically basal species (*N.cinnabarina*, *N.didymospora*, and *N.microspora*) tend to have cylindrical, oblong, and longer conidia than the spherical conidia of the terminal species (*N.acutata*, *N.multiguttata*, and *N.obtusa*). Germination was only observed in the long conidia of *N.cinnabarina* and *N.microspora*, suggesting that the spherical conidia of the terminal species lack germination ability and are associated with sexual reproduction as spermatia rather than dispersal (Higgins 1920; Drayton 1932). Asexual stages of Mollisiaceae are sometimes produced after prolonged incubation at cool temperatures (Tanney et al. 2016), which might be required to assess asexual stages in future descriptions. We believe that more detailed and precise species description can be achieved by combining much more characteristics, including sexual and asexual stages, hyphal structures, and host selectivity.

### ﻿Host preference and distribution

Half of the new fungal species described in this study were collected from bamboos (including bamboo grasses) and *Miscanthussinensis*. Various species of bamboos and *Miscanthus* are widely distributed from subtropical to temperate regions, except Europe, suggesting that the center of speciation is in East Asia (Takeda 1988; Nishiwaki and Nadir 2014). In Japan, bamboos are widely distributed, and its species and varieties are remarkably diverse. Hino and Katumoto (1961) focused on the diversity of bamboos in Japan and discovered an astonishing number of new bambusicolous fungi including *B.longispora* (126 species in 10 genera). As most existing species of *Belonopsis* and *Trichobelonium* have been found in Europe from non-bambusicolous host, more species associated with endemic *Miscanthus* spp. and bamboos are expected in East Asia.

## ﻿Conclusion

Most species of mollisioid fungi have been described in Europe and its species diversity in Japan has been largely overlooked. In this study, we described nine species in *Neobelonopsis* gen. nov. and two new species in *Trichobelonium* based on morphology, ecology, and phylogenetic analysis. This study also indicated that more undescribed species of mollisioid fungi will be discovered by exploration focusing on the substrates characteristic of East Asia.

To support generic distinction of *Neobelonopsis* from *Belonopsis*, *Mollisia*, and *Trichobelonium*, DNA sequencing data are wanted. It is also possible that some species currently classified in *Belonopsis* or *Trichobelonium* would be transferred to *Neobelonopsis* by further phylogenetic analysis. Therefore, the phylogenetic placement of the type species of *Trichobelonium*, *T.kneiffii* must be resolved and additional sequencing of *Belonopsis* and *Trichobelonium* spp. is required.

## Supplementary Material

XML Treatment for Neobelonopsis

XML Treatment for Neobelonopsis acutata

XML Treatment for Neobelonopsis bicolor

XML Treatment for Neobelonopsis cinnabarina

XML Treatment for Neobelonopsis didymospora

XML Treatment for Neobelonopsis microspora

XML Treatment for Neobelonopsis multiguttata

XML Treatment for Neobelonopsis obtusa

XML Treatment for Neobelonopsis ramosa

XML Treatment for Trichobelonium albobarbatum

XML Treatment for Trichobelonium miscanthi

XML Treatment for Neobelonopsis diesbachiana

## ﻿Additional information

### Author ORCIDs

Hiyori Itagaki https://orcid.org/0000-0001-8678-0826

Tsuyoshi Hosoya https://orcid.org/0000-0001-5360-5677

### Data availability

All of the data that support the findings of this study are available in the main text.
